# Expression of Linear and Novel Circular Forms of an *INK4/ARF*-Associated Non-Coding RNA Correlates with Atherosclerosis Risk

**DOI:** 10.1371/journal.pgen.1001233

**Published:** 2010-12-02

**Authors:** Christin E. Burd, William R. Jeck, Yan Liu, Hanna K. Sanoff, Zefeng Wang, Norman E. Sharpless

**Affiliations:** 1 The Curriculum in Toxicology, The Lineberger Comprehensive Cancer Center, The University of North Carolina School of Medicine, Chapel Hill, North Carolina, United States of America; 2 Department of Genetics, The University of North Carolina School of Medicine, Chapel Hill, North Carolina, United States of America; 3 The Division of Hematology and Oncology, University of Virginia, Charlottesville, Virginia, United States of America; 4 Department of Pharmacology, The University of North Carolina School of Medicine, Chapel Hill, North Carolina, United States of America; 5 Department of Medicine, The University of North Carolina School of Medicine, Chapel Hill, North Carolina, United States of America; Stanford, United States of America

## Abstract

Human genome-wide association studies have linked single nucleotide polymorphisms (SNPs) on chromosome 9p21.3 near the *INK4/ARF (CDKN2a/b)* locus with susceptibility to atherosclerotic vascular disease (ASVD). Although this locus encodes three well-characterized tumor suppressors, *p16^INK4a^*, *p15^INK4b^*, and *ARF*, the SNPs most strongly associated with ASVD are ∼120 kb from the nearest coding gene within a long non-coding RNA (ncRNA) known as *ANRIL* (*CDKN2BAS*). While individuals homozygous for the atherosclerotic risk allele show decreased expression of *ANRIL* and the coding *INK4/ARF* transcripts, the mechanism by which such distant genetic variants influence *INK4/ARF* expression is unknown. Here, using rapid amplification of cDNA ends (RACE) and analysis of next-generation RNA sequencing datasets, we determined the structure and abundance of multiple *ANRIL* species. Each of these species was present at very low copy numbers in primary and cultured cells; however, only the expression of *ANRIL* isoforms containing exons proximal to the *INK4/ARF* locus correlated with the ASVD risk alleles. Surprisingly, RACE also identified transcripts containing non-colinear *ANRIL* exonic sequences, whose expression also correlated with genotype and *INK4/ARF* expression. These non-polyadenylated RNAs resisted RNAse R digestion and could be PCR amplified using outward-facing primers, suggesting they represent circular RNA structures that could arise from by-products of mRNA splicing. Next-generation DNA sequencing and splice prediction algorithms identified polymorphisms within the ASVD risk interval that may regulate *ANRIL* splicing and circular *ANRIL* (*cANRIL*) production. These results identify novel circular RNA products emanating from the *ANRIL* locus and suggest causal variants at 9p21.3 regulate *INK4/ARF* expression and ASVD risk by modulating *ANRIL* expression and/or structure.

## Author Summary

Unbiased studies of the human genome have identified strong genetic determinants of atherosclerotic vascular disease (ASVD) on chromosome 9p21.3. This region of the genome does not encode genes previously linked to ASVD, but does contain the *INK4/ARF* tumor suppressor locus. Products of the *INK4/ARF* locus regulate cell division, a process thought to be important in ASVD pathology. We and others have suggested that genetic variants in 9p21.3 influence *INK4/ARF* gene expression; however, the mechanisms by which these distant polymorphisms (>100,000 bp away) influence transcription of the locus is unknown. The ASVD–associated genetic variants lie within the predicted structure of a non-coding RNA (ncRNA) called *ANRIL*. Based upon recent work suggesting that other ncRNAs can repress nearby coding genes, we considered the possibility that *ANRIL* structure may regulate *INK4/ARF* gene expression. Coupling molecular analysis with state-of-the-art sequencing technologies in a wide variety of cell types from normal human donors and cancer cells, we found that *ANRIL* encodes a heterogeneous species of rare RNA transcripts. Moreover, we identified novel, circular *ANRIL* isoforms (*cANRIL*) whose expression correlated with *INK4/ARF* transcription and ASVD risk. These studies suggest a new model wherein *ANRIL* structure influences *INK4/ARF* expression and susceptibility to atherosclerosis.

## Introduction

Atherosclerotic vascular disease (ASVD) is a leading cause of human mortality worldwide [1]. While there are well-recognized risk factors for ASVD such as tobacco use, obesity and hyperlipidemia, the identification of common genetic variants associated with the disease has proven difficult despite strong evidence that susceptibility is heritable. Recently, multiple unbiased genome-wide wide association studies (GWAS) have linked single nucleotide polymorphisms (SNPs) on chromosome 9p21 to ASVD and other related conditions (*i.e.* coronary artery disease, stroke, myocardial infarction and aortic aneurysm) [2]–[12]. These associations have been replicated in multiple independent studies and are not associated with “classical” ASVD risk factors such as hypertension, obesity, tobacco use or lipid levels. While causal variants within 9p21.3 have yet to be identified, the risk associated SNPs cluster together within a 53 kb region roughly 100 kb centromeric to the *INK4/ARF* (*CDKN2a/b*) tumor suppressor locus ([13] and Figure S1). Congenic mapping using mice differentially susceptible to ASVD has identified syntenic susceptibility alleles near the murine *Ink4/Arf* locus [14], suggesting that this risk interval is conserved in mammals.

The *INK4/ARF* locus encodes three archetypal tumor suppressor genes: *p16^INK4a^*, *p15^INK4b^* and *ARF* (*p14^ARF^* in humans, *p19^ARF^* in mice), as well as a long non-coding RNA called Antisense Non-coding RNA in the *INK4* Locus (*ANRIL* or *CDKN2BAS*). Other than a role for ARF in development of the optic vasculature, all of the *INK4/ARF* proteins are thought to be largely dispensable for normal mammalian development, but play important roles in restraining aberrant proliferation associated with cancer and other disease states (reviewed in: [15]). In purified T-cells from healthy individuals, we have shown a pronounced effect of ASVD-associated SNPs at 9p21 on *INK4/ARF* expression, with those harboring the risk alleles demonstrating reduced levels of *p15^INK4b^*, *p16^INK4a^*, *ARF* and *ANRIL* [16]. Decreased expression of such anti-proliferative molecules could promote pathologic monocytic or vascular proliferation, thus accelerating ASVD development (reviewed in [17], [18]). For example, mice lacking *p16^INK4a^* exhibit increased vascular hyperplasia following intra-arterial injury [19] and *ARF* deficiency has been implicated in atherosclerotic plaque formation [20]. Additionally, TGF-β signaling, which induces the expression of *p16^INK4a^* and *p15^INK4b^*, is anti-atherogenic in some settings [21]–[23]. Most recently, excess proliferation of hematopoietic progenitor cells, which is in part controlled by *p16^INK4a^* expression during aging [24], has been associated with atherosclerosis in a murine model [25]. Moreover, targeted deletion of a region syntenic to the ASVD risk interval in mice resulted in severely attenuated expression of *p15^INK4b^* and *p16^INK4a^* [26]. Although these results suggest that the ASVD-associated 9p21 SNPs control *INK4/ARF* expression, and that decreased expression of the *INK4/ARF* tumor suppressors may promote ASVD, it is not known how polymorphisms located ∼120 kb away from the locus might influence *INK4/ARF* expression.

*ANRIL* was first uncovered in a genetic analysis of familial melanoma patients with neural system tumors [27]. Based upon EST assembly, *ANRIL* has 19 exons with no identified open reading frame [27] (Figure S2). Although cloning a full-length version of the predicted transcript has proven difficult, a growing number of alternatively spliced *ANRIL* transcripts have recently been reported in the literature [28], [29]. Many of these reports suggest that multiple *ANRIL* isoforms can be expressed in a single cell type. For example, two *ANRIL* variants have been reported in testes, five in HUVECs and three in lung [27], [28]. Further confounding the study of *ANRIL*, the majority of predicted exons are <100 nucleotides in length and many consist entirely of repetitive LINE, SINE and *Alu* elements (*i.e.* exons 7, 8, 12, 14 and 16) [30]. Without a firm understanding of *ANRIL* structure, deciphering the biological function of this non-coding RNA has become increasingly complicated.

More than a decade ago, elegant genetic work by Van Lohuizen, DePinho and colleagues demonstrated that the *Ink4/Arf* locus is potently repressed by Polycolmb group (PcG) complexes [31]. Such repression appears critical for the persistence and proliferation of somatic stem cells and other self-renewing tissues such as pancreatic beta-cells [32], [33]. Until recently little progress was made in understanding the biochemical basis for PcG-mediated *INK4/ARF* silencing, however two independent groups have since shown that the PcG complexes, PRC-1 and PRC-2, localize to the *INK4/ARF* locus and repress its activity through the establishment of repressive chromatin modifications such as H3K27 trimethylation [34]–[36]. Prior work has also shown that long, non-coding RNAs such as *Xist*, *Kcnq1ot1* and *HOTAIR* can repress genes in *cis*- or trans*-* through interaction with PcG complexes [37]–[40]. Moreover, dysregulation of *HOTAIR* has been recently implicated in breast cancer progression, suggesting that long, non-coding RNAs play an important role in human disease [41]. Based on this evidence, we and others postulated that *ANRIL* could play a similar role in PcG-mediated repression of the *INK4/ARF* locus [16].

Against this background of prior work, we sought to better determine *ANRIL* structure and expression in relation to ASVD-SNP genotype and *INK4/ARF* expression. Toward that end, we performed comprehensive DNA and RNA analyses of *ANRIL* using RACE and next-generation sequencing in primary and transformed cell lines. We showed that the expression of *ANRIL* isoforms containing exons 1–2 or 4–6 correlated with ASVD-SNP genotype, however those containing exons 18–19 did not. Surprisingly, we also uncovered circular *ANRIL* (*cANRIL*) RNA species whose expression correlated with ASVD-SNP genotype. These new data suggest that the causal variant(s) within 9p21 influence ASVD susceptibility through regulation of *ANRIL* expression and splicing, leading to differential PcG recruitment and *INK4/ARF* repression.

## Results

### *ANRIL* transcription produces multiple rare, non-coding RNA species

**Figure 1:**
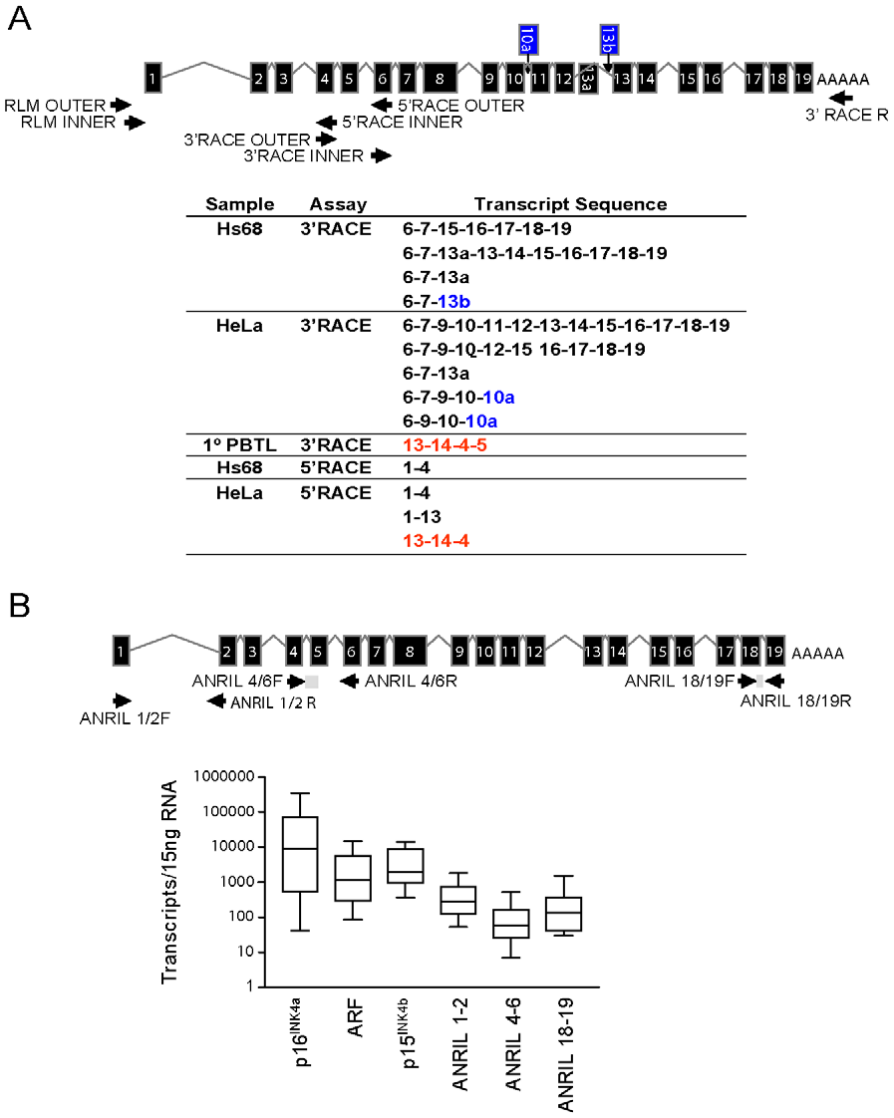
Identification and characterization of *ANRIL* splice variants. A, 3′ and 5′ RACE was performed using primers directed against exons 4 and 6 where long stretches of unique sequence were observed (top). The resulting PCR products were cloned and sequenced, revealing several novel exons shown in blue (10a and 13b) and multiple non-colinear species (red). B, Equal quantities of total RNA were harvested from growing cell lines of various tissue types and absolute expression of the indicated transcript was determined. Expression levels are shown in a box-whisker plot on a log_10_ scale in 11 of 27 analyzed cell lines which did not harbor homozygous 9p21 deletion. Validated Taqman detection strategies for the indicated *ANRIL* species are shown (top).

Multiple *ANRIL* isoforms have been proposed based upon the assembly of ESTs and the sequencing of cDNA libraries (See Figure S2). To determine which of these isoforms predominates *in vivo*, we performed RNA ligase mediated (RLM)-RACE in cell lines and primary human peripheral blood T-lymphocytes (PBTL). In addition to using the RLM procedure to maximize the detection of mRNA transcripts, we employed a high-fidelity Taq polymerase capable of amplifying complex DNAs such as those containing *SINE*, *LINE* and *Alu* elements. Primers for 3′ and 5′ RACE were designed within exons 1, 2, 4, 6, 9, 13, 16 and 18 of the originally reported transcript, NR_003529 [27], but only primers in exons 4 and 6 selectively amplified *ANRIL* sequences (data not shown). These amplicons were cloned and sequenced to verify the resulting DNA sequences. Using exon 4 and 6 primers, we identified multiple *ANRIL* variants, including novel splice isoforms that were not previously reported (Figure 1A, novel exons shown in blue). We also detected several transcripts with a peculiar, non-colinear exon sequence, most notably in the HeLa and primary PBTL populations (shown in red in Figure 1A). Given that no particular *ANRIL* isoform appeared to predominate from our RACE data, we exploited the observation that *ANRIL* RNAs containing exon 15 frequently maintained the canonical exonic structure (*e.g.* 15–16–17–18–19). We termed these exons as “distal” because they are located at the 3′ end of *ANRIL*, and those prior to exon 15 as “proximal”.

To examine the absolute abundance of transcripts containing the “proximal” or “distal” exons in a variety of cell types, we developed and validated quantitative Taqman primer-probe sets spanning *ANRIL* exons 1–2, 4–6 and 18–19 (See Materials and Methods and Figure 1B). We also quantified the expression of other *INK4/ARF* transcripts *(e.g. p15^INK4b^*, *p16^INK4a^* and *ARF*) since we previously observed a correlation between the expression of *ANRIL4-6* and *p16^INK4a^* in PBTLs (r^2^ = 0.32, p<0.0001; [42]). Importantly, none of these Taqman strategies amplify *ANRIL* exons containing repetitive sequences. Moreover, the products from these assays were gel-purified, cloned and sequenced to verify their identities. Equal amounts of RNA from a panel of 27 primary and transformed cell lines were subjected to reverse transcription using a combination of random hexamer and oligo dT primers. Deletion or methylation of the 9p21 tumor suppressors is a common event in cancer and was observed in 59% of our cell line panel (Figure S4). Even after exclusion of these lines from our analyses, all three *ANRIL* species (exon *1*–*2*, *4*–*6* and *18*–*19*) were rare: based on an estimate of 15 pg of total RNA per cell, expression of *ANRIL* species ranged between 0.01 to 1 copy per cell (Figure 1B). For comparison, *INK4/ARF* expression is considered quite low in primary cells [43], [44], yet *p16^INK4a^* and *p15^INK4b^* were more abundant than any *ANRIL* species in non-transformed Hs68, HUVEC and IMR90 cell lines (on average 155.9±18.9- and 14.5±5.5-fold, respectively; See Figure S4). These data indicate that *ANRIL* species are readily detectable but non-abundant in a wide variety of cell types.

### Central *ANRIL* exons are weakly detected in cell line and RNA-seq analyses

**Figure 2:**
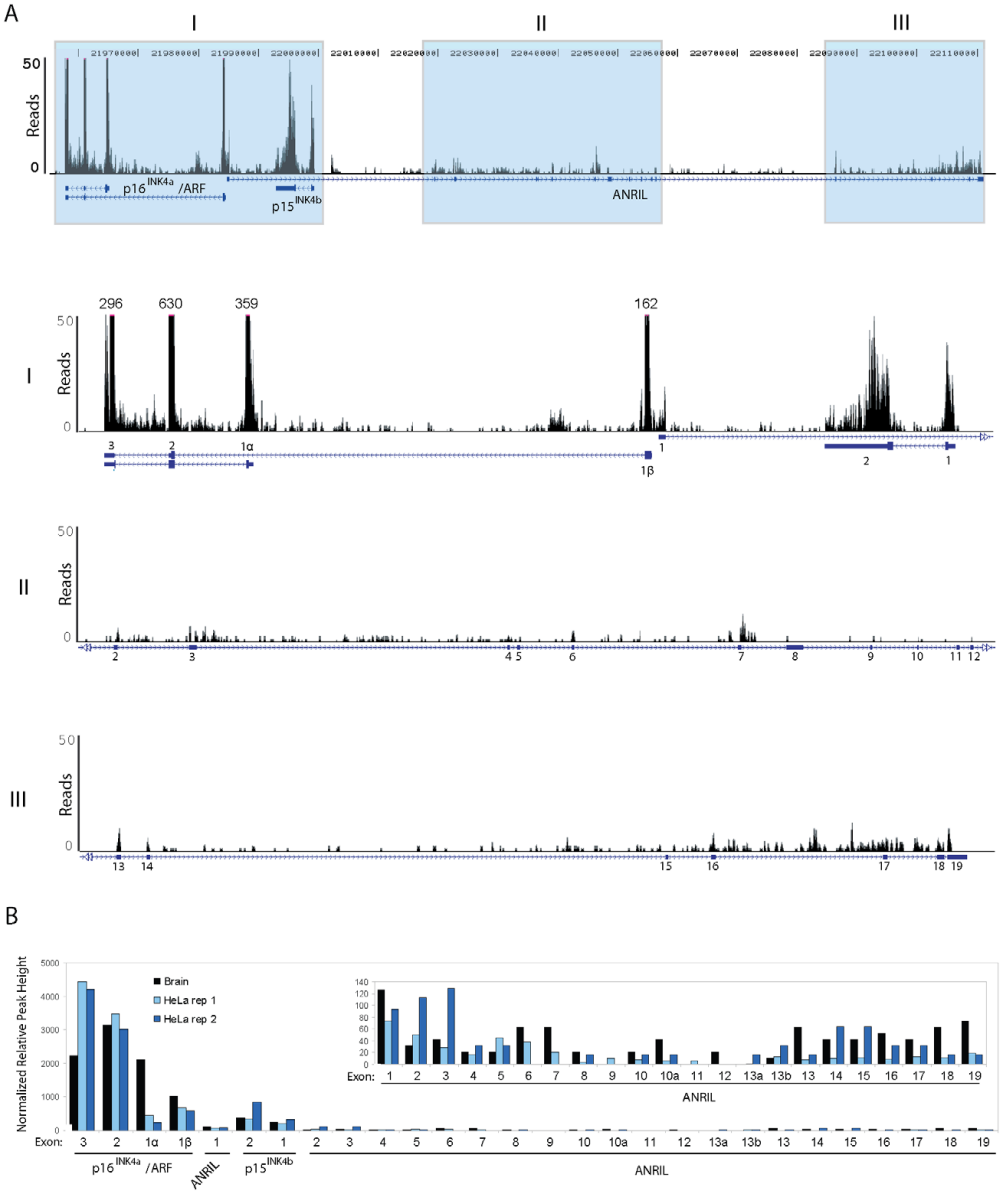
RNA Sequencing of *ANRIL* transcripts. A, Coverage plots of RNA sequencing reads derived from short read archive study SRP002274 [45]. *Top*, Read coverage across all *ANRIL* exons and nearby tumor suppressor genes *p16^INK4a^*, *ARF* and *p15^INK4b^* is shown. *Bottom*, The grey regions in the top panel were graphed on a truncated scale to better depict *ANRIL* coverage. Annotations above the larger peaks show the maximum number of reads mapping to these areas. B, Maximum peak height at each exon (normalized by overall locus coverage) is displayed from three independent samples: SRP002274 (Brain) and two ENCODE RNA-sequencing replicates of the HeLa cell line (HeLa rep 1 and 2). The inset shows all *ANRIL* exons on y-axis with peak height of 150 reads.

To further characterize 9p21 transcription in a manner unbiased by PCR primer choice, we analyzed publically available, next-generation RNA sequencing (RNA-seq) datasets of oligo dT-primed and reverse transcribed RNA from primary human brain or HeLa cells. ([45]; Genbank Short Read Archive Study ID: SRP002274 and ENCODE RNA-seq replicates, respectively). These datasets were solely chosen based upon the high-depth of sequencing provided (>368 million and >110 million reads, respectively), allowing for the study of non-abundant ncRNAs. In accord with the TaqMan-based analyses, reads mapping to *ANRIL* were rare in both the human brain and HeLa datasets (Figure 2). In fact, the peak height of reads mapping to *p16^INK4a^* was >40 times higher than that of any *ANRIL* exon, even though brain expresses low levels of p16^INK4a^ [44], [46]. The level of *ANRIL* expression detected in both datasets (maximal peak height of 12) was comparable to that of other long, non-coding RNAs including *HOTAIR* and *Kcnq1ot1* (maximal peak heights of 10 and 18, respectively).

Intriguingly, by both TaqMan (Figure 1B) and RNA-seq (Figure 2B), we observed a disparity in the number of molecules of each *ANRIL* exon detected, with those containing the ‘central’ *ANRIL* (4–12) exons being the least abundant when averaged across the entire locus. The most prevalent *ANRIL* peaks localized to the 5′ and 3′ ends of the transcript (*i.e.* exons 1–3 and 13–19). The relative excess of the more distal exons might be a consequence of 9p21 deletion, as we observed splicing between *MTAP*, a gene 100 kb telomeric to the *INK4/ARF* locus, and the 3′ end of *ANRIL* in RNA-seq datasets from cell lines with *INK4/ARF* loss (*i.e.* SUM102 and MCF7, data not shown). Although such *MTAP*-*ANRIL* fusions have been previously described [47], using a highly sensitive Taqman strategy we only detected these fusions in cell lines with 9p21 deletion, and not in any primary cells or lines with two copies of an intact *INK4/ARF* locus (data not shown). Therefore, while exon deletions and/or *MTAP* splicing explain the relative excess transcription of distal *ANRIL* exons in some cancer cell lines harboring 9p21 deletion, these mechanisms do not explain the decreased transcription of exons 4–12 versus exons 1–3, in any cell line. Likewise, somatic deletions and *MTAP* splicing do not explain the uniform decrease of the central exons compared to the proximal and distal exons in cultures of primary cells (Figure S4). Together, these RNA-seq and TaqMan analyses indicate that *ANRIL* is a rare, multi-variant RNA species in which transcripts containing exons 1–3 or 13–19 predominate over those containing exons 4–12. Based on these findings as well as the observation of non-colinear RNA species by RACE (Figure 1A), we hypothesized that the reduced level of the central exons (4–12) in mature, polyadenylated RNA may be the result of alternative splicing events in which the central exons of *ANRIL* are frequently skipped.

### Select *ANRIL* transcripts containing internal exons are circular

We observed non-colinear RNA species in RACE analysis of several cell lines and primary cells (Figure 1A). We considered the possibility that this represented alterations of the germline DNA sequence (*e.g.* duplications or transversions) but found no evidence for this using BreakDancer [48] to analyze next-generation DNA sequencing from 10 individuals. Given that cryptic germline DNA alterations did not appear to explain these non-colinear forms, we considered that non-canonical RNA splicing events such as *trans-*splicing or re-splicing of RNA lariats might occur as part of *ANRIL* processing. In the latter case, the splice sites of certain consecutive exons are not recognized by the splicing machinery, resulting in the inclusion of exonic sequences within the RNA lariat [49]. Internal splicing of this exon-containing lariat structure may then occur, leading to the production of exon-only RNA circles in which non-consecutive exon junctions are generated by splicing across the branch site (*e.g*. exon 13–14–4–5).

**Figure 3:**
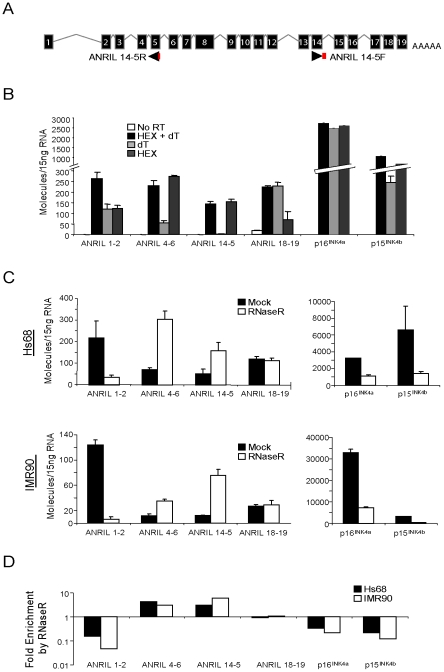
*ANRIL 14-5* and *4-6* are circular RNAs. A, Schematic representation of the *ANRIL14-5* Taqman detection strategy wherein the probe (red) spans the exon 5-exon 14 boundary amplified with outward facing primers. B, Expression of the indicated transcripts was quantified in cDNA from Hs68 cells made using the indicate primers (-RT: no reverse transcriptase, H+dT: an equal mix of random hexamers and oligo dT, dT: oligo dT alone, and HEX: random hexamers alone). Error bars represent the standard deviation for three replicates. C, Total RNA harvested from growing Hs68 (top) and IMR90 (bottom) cells was incubated with our without RNase R, purified and reverse transcribed. The indicated transcripts were quantified in ‘B’. D, The average fold enrichment by RNase R for each transcript is shown on a log_10_ scale.

To determine the structure of such non-colinear *ANRIL* RNAs, we designed and validated a Taqman strategy using outward facing primers to detect transcripts containing the non-colinear exon 14-5 junction (Figure 3A). This detection scheme was chosen based upon preliminary PCR studies wherein amplification of the 14-5 splice junction predominated over that of the exon 14-4 junction. As lariat or circular RNAs would not be polyadenylated, we assayed the amount of each *ANRIL* species detected in RNA samples reverse transcribed with random hexamers (HEX), oligo dT (dT) or both (Figure 3B). As would be expected for polyadenylated RNAs, conversion of *ANRIL1-2*, *ANRIL18-19*, *p16^INK4a^* and *p15^INK4b^* into cDNA was efficiently accomplished with either oligo dT or HEX primers alone. Conversely, *ANRIL4-6* and *14-5* were effectively primed with HEX but not oligo dT, confirming that these transcripts were not polyadenylated.

To further examine the structure of these non-canonical transcripts, we determined transcript sensitivity to the RNA exonuclease, RNAse R. RNAse R specifically digests both structured and non-structured linear RNAs, but spares RNA circles and lariats [50]. Using equal amounts of RNA from normal and immortalized human fibroblasts (IMR90 and Hs68, respectively) treated with or without RNAse R, we generated cDNA and conducted TaqMan analysis for *ANRIL* expression. As expected for linear species, RNAse R treatment caused a marked reduction in the number of *p16^INK4a^* and *p15^INK4b^* transcripts detected (4.5- and 8.4-fold decrease, respectively), demonstrating that these coding transcripts are predominantly linear (Figure 3C and 3D). In contrast, we observed a 6-fold enrichment of *ANRIL14-5* molecules with RNAse R treatment, confirming their circular nature. We were surprised to find that *ANRIL4-6* expression also exhibited RNAse R-dependent enrichment in both cell lines. *ANRIL1-2* levels decreased consistent with a predominantly linear species, and *ANRIL18-19* demonstrated an intermediate behavior consistent with a mix of linear and circular forms (Figure 3C and 3D). Together, these data provide evidence that *ANRIL4-6* and *14-5* are predominantly contained within non-polyadenylated, circular (or lariat) *ANRIL* (*cANRIL*) transcripts.

### Circular *ANRIL* species are observed in a wide variety of cell types

Based upon analysis of non-sequential cDNA and EST sequences, stable RNA circles have been hypothesized to represent <1% of the total transciptome [51]. To determine the ubiquity of *cANRIL* RNAs, we employed the Taqman-based strategy shown in Figure 3A. Using this method, we observed amplification of *ANRIL14-5* in 16 of 20 *ANRIL* expressing primary cultures and cell lines (Figure S5). The finding of *cANRIL* in diverse cell types is perhaps surprising given the low abundance of *ANRIL* transcripts and that RNA lariats are usually unstable intermediates which undergo rapid degradation. These data suggest that *cANRIL* is a stable, naturally occurring, circular RNA species produced in most *INK4/ARF* expressing cells.

**Figure 4:**
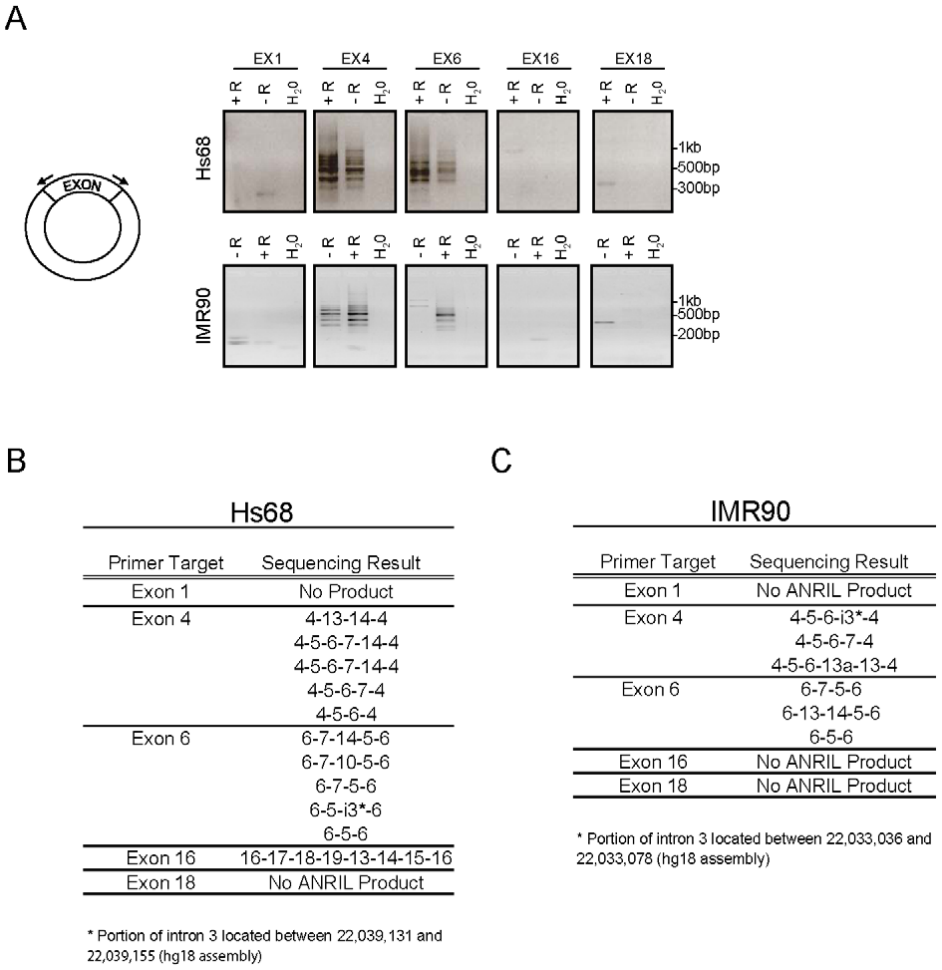
*ANRIL* circular RNAs predominantly contain exons 4-14. A, cDNA generated in the presence (+R) or absence (-R) of RNase R as in Figure 3C was subjected to PCR using outward facing primers within the same exon as depicted (left) and separated by gel electrophoresis. B and C, The PCR products in ‘A’ were purified, cloned and sequenced. The resulting sequences are shown for each exon pair.

To better define the structure of *cANRIL* RNAs, we used outward-facing primers located in five *ANRIL* exons to amplify cDNA produced using a mix of random hexamers and oligo dT (Figure 4A). In Hs68 and IM90 cells, PCR products specific to 9p21 were detected using primers sets targeting exons 4 and 6. These products were more efficiently amplified using cDNAs from RNase R-treated samples, suggesting that they arose from circular RNAs. In contrast, PCR products specific to chromosome 9 were not detected using primers internal to exons 1 and 18, and only detected in one instance with primers internal to exon 16, suggesting these exons were not commonly included in stable, circular *ANRIL* species. To determine the contents of the circular transcripts containing *ANRIL* exons 4 and 6, we cloned and sequenced the observed PCR products. The resulting sequences predominantly included *ANRIL* exons 4 to14. Several exons were never observed in the looped structures (*i.e.* exons 1, 2, 3, 8, 9, 11, and 12) (Figure 4B and 4C). Novel sequences were also discovered within the products, most notably, regions intronic to the previously described *ANRIL* transcript (*i.e.* parts of intron 3). These data explain the paucity of expression of *ANRIL* exons 4 to 14 as determined by Taqman and RNA-seq (Figure 1B and Figure 2B), suggesting that *ANRIL* processing may involve exon skipping events which preferentially incorporate the central exons into lariats which may then be internally spliced to form *cANRIL*.

### Expression of *cANRIL* and proximal *ANRIL* species correlate with *INK4/ARF* expression and ASVD–associated SNP genotype

**Figure 5:**
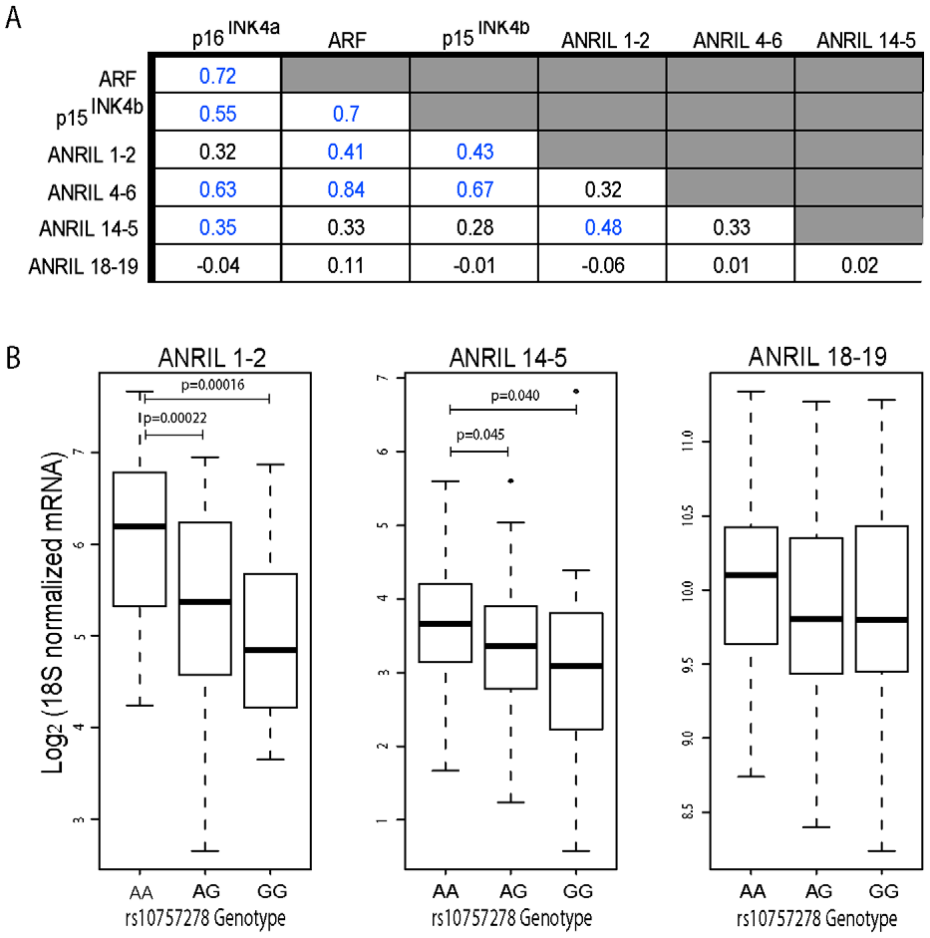
*ANRIL4-6* and *14-5* correlate with *INK4/ARF* expression and rs10757278 genotype in human PBTLs. A, Taqman anlaysis of *ANRIL* and *INK4/ARF* transcripts was conducted and normalized as described in Materials and Methods. Correlations between *ANRIL* and *INK4/ARF* transcripts in 106 primary peripheral blood T-lymphocytes (PBTLs) were determined using linear regression. The R value for each pair-wise comparison is shown, with those achieving significance (p<0.05) depicted in blue. Due to limitations in sample availability, *ANRIL 1-2* and *ANRIL 14-5* levels were determined in only a subset of individuals (n = 94 and 98, respectively). B, The relative expression of *ANRIL1-2*, *14-5* and *18-19* normalized as in ‘A’ is plotted versus rs10757278 genotype. p-values were determined by a two-sided t-test.

We have previously shown [42] a correlation between expression of the coding *INK4/ARF* transcripts and *ANRIL4-6*. We now recognize that the latter is largely contained in circular RNA species (Figure 3B and 3C). Next, we investigated correlations between other, newly identified *ANRIL* isoforms and the coding *INK4/ARF* transcripts (Figure 5A and Figure S6). We performed Taqman-based gene expression analyses in 106 primary human peripheral blood T-lymphocyte (PBTL) samples from two previously published cohorts [16], [42]. Remarkably, *cANRIL* expression (*ANRIL14-5* and *ANRIL4-6*) was observed in all PBTL samples. Expression of *ANRIL18-19* did not correlate with any other *ANRIL* or *INK4/ARF* transcript (Figure 5A). In contrast, a strong correlation was observed between the expression of *ANRIL4-6* and all of the *INK4/ARF* tumor suppressors (p<1*10^−15^ for all pair-wise comparisons). Significant associations (p<0.05) were also observed between *ANRIL1-2* and *ARF*, and between *ANRIL14-5* and *p16^INK4a^*.

We have also demonstrated an effect of a replicated SNP associated with atherosclerosis (rs10757278) on the expression of *p15^INK4b^*, *p16^INK4a^*, *ARF* and *ANRIL4-6* in PBTLs [16]. We therefore examined whether expression of other *ANRIL* isoforms correlated with ASVD SNP genotype (Figure 5B). While no significant correlation between *ANRIL18-19* with SNP genotype was noted, expression of *ANRIL1-2* and *ANRIL14-5* showed significantly decreased expression in individuals harboring the risk (G) allele (p<0.0001 and p<0.04, respectively). In aggregate, these data demonstrate that expression of both circular and linear transcripts containing the proximal but not distal *ANRIL* exons correlates with ASVD-SNP genotype and expression of the coding *INK4/ARF* transcripts.

### Deep sequencing of the ASVD risk interval reveals *ANRIL* exon 15 variants predicted to influence c*ANRIL* production

**Figure 6:**
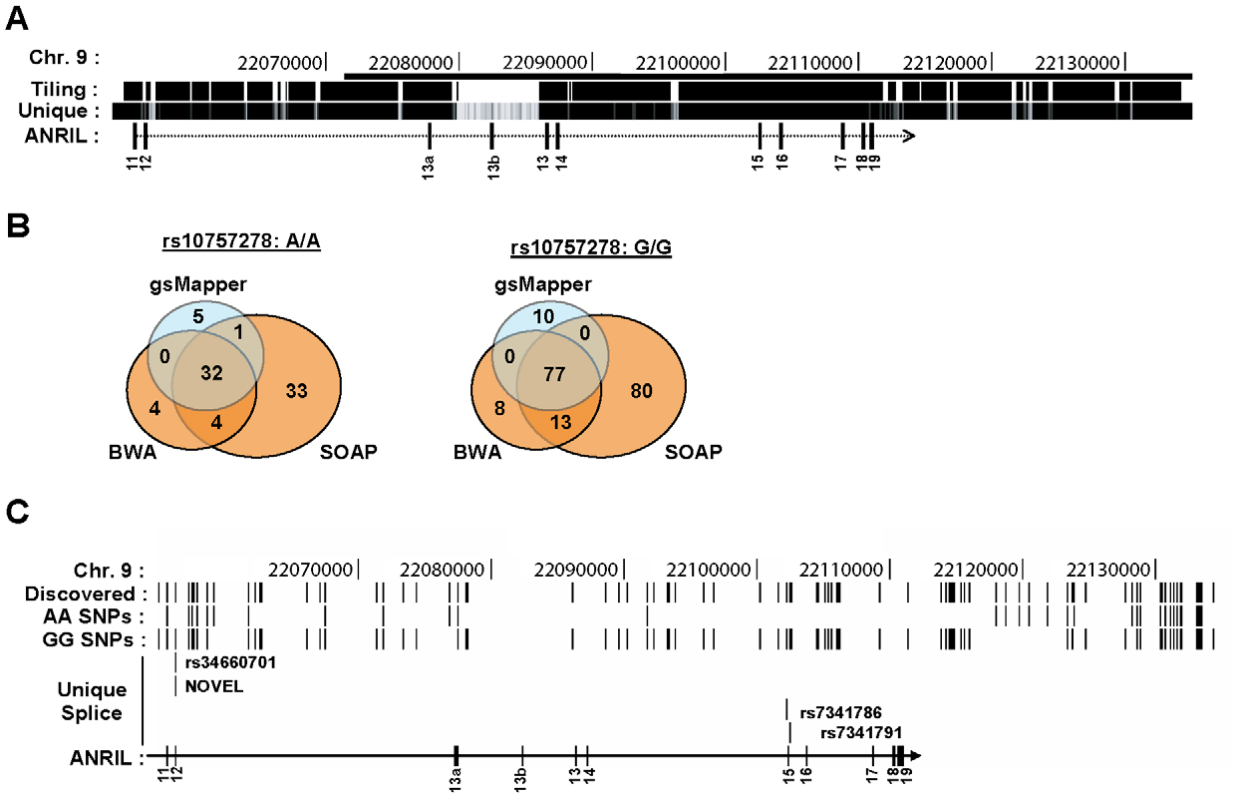
Deep sequencing of 9p21 in pools of rs10757278 homozygotes. A, The region captured using DNA sequence capture technology is shown on the ‘Tiling’ track. The ‘Unique’ track shows the Duke 35 bp Uniqueness information as provided in the UCSC Genome Browser. The bar at the top of the figure represents the 53 kb risk interval previously defined by Broadbent *et al.* [13]. B, Venn diagrams depicting SNP calls for the AA (left) and GG (right) samples using three different algorithms. C, Using the UCSC Genome Browser, SNPs identified by next-generation DNA sequencing are depicted across the captured region of 9p21. The ‘Discovered’ track shows the polymophisms identified by two or more algorithms in either the pooled AA or GG sample. SNPs unique to each genotype are shown below. The ‘Unique Splice’ track depicts the location of the 4 SNPs, unique to the GG sample, which modify *cis*-acting splice regulation sites (See also Table S1).

We performed next-generation DNA sequencing of the ASVD risk interval to identify polymorphisms that might influence *ANRIL* expression or splicing. To enhance detection of rare variants, we pooled DNA from 5 individuals of Asian and European descent who were homozygous for either the A or G allele of rs10757278. To increase the chance that the analyzed DNAs would harbor causal variants that influence *INK4/ARF* expression, we selected individuals based on their age-adjusted expression of *p16^INK4a^* (*i.e.* AA donors with higher than average expression, GG donors with lower than average expression [16]). We performed sequence capture using an Illumina tiling array on the AA vs. GG pooled samples. The tiling array was designed to bind all non-repetitive human chromosome 9 regions from 22,054,888 to 22,134,171 bp, a region chosen as it contains the previously identified ASVD and type 2 diabetes mellitus risk intervals (Figure 6A). The 9p21 enriched DNA was sequenced using both Roche 454 (400 bp reads) and Illumina GAII (35 bp reads) technologies. The resulting sequences were aligned to the UCSC reference genome (hg18) using three separate alignment algorithms: gsMapper, BWA and SOAP [52], [53]. As shown in Figure 6B, SOAP identified the highest number of polymorphisms within each pooled sample; however, many of these were not recapitulated using other mapping techniques. Thus, for further analysis we focused our efforts on polymorphisms which were identified by at least two mapping algorithms. Employing these criteria, we discovered 101 SNPs that differed from the reference genome within our samples: 11 in the AA pool, 64 in the GG pool, and 26 in both (Figure 6C). As expected, more differences from the reference genome were identified in the GG sample, as the reference genome harbors the A allele, and most SNPs in the captured region are in moderate to strong linkage disequilibrium with rs10757278 (Figure S1). Therefore, using next generation sequencing of captured DNA from 10 informative individuals biased to harbor causal variants, the chosen sequencing approach found 75 (11+64) SNPs that differed between the two pooled samples.

Given the finding that *cANRIL* expression correlates with rs10757278 genotype, we sought to determine if any of the identified SNPs might influence *ANRIL* splicing. For this analysis, we restricted the SNP list to include only those within 200 bp of an *ANRIL* intron-exon boundary, as genetic alterations in this region have the highest potential to influence RNA splicing [54]. Of the 75 SNPs present in only one of the two pooled samples, four were within 200 bp of an *ANRIL* intron-exon boundary (Figure 6C, Table S1). Using previously described prediction algorithms [54], [55], we determined the likelihood of these variants near intron-exon boundaries to change *cis*-regulatory splice elements including exon splicing enhancers (ESEs) and silencers (ESSs) as well as intronic splicing enhancers (ISEs) and silencers (ISSs). A score of -1 indicates that the minor allele destroys one *cis*-element and +1 indicates that the minor allele creates one *cis*-element (Table S1). ESEs and ISEs favor exon recognition by the spliceosome when they occur in exons or introns, respectively. However, ISSs and ESSs promote exon skipping irrespective of position [56]. A pair of SNPs (rs34660702 and NOVEL) 10 bp apart near the start of exon 12 were predicted to alter ANRIL splicing, but the effects of these SNPs worked in opposite directions, and both exhibited low frequencies in the CEU population (Figure 6C and Table S1). More provocatively, we also observed two SNPs (rs7341786 and rs7341791) in strong linkage disequalibrium with rs10757278 and which bracketed exon 15. These SNPs demonstrated high minor allele (CC and GG, respectively) frequencies and with the major allele of both SNPs enhancing the “exon-ness” of exon 15. These results indentify common, linked SNPs whose major allele is likely to inhibit skipping of exon 15 and thereby promote the production of *cANRIL* species ending in exon 14 in individuals homozygous for the A (protective) allele of rs10757278.

Due to the small number of pooled individuals for sequencing (n = 5/group), we also sought to identify additional, rare polymorphisms with the potential to modulate *ANRIL* splicing. We analyzed all SNPs reported in the HapMap database in the chromosome 9 region 22,054,888 to 22,134,171 within 200 bp of *ANRIL* intron-exon boundaries for their potential to alter RNA splicing in *cis-*. Using this method, we identified 31 additional SNPs in HapMap that modified ESE, ESS, ISE or ISS sequences (Table S1). Notably, the majority of these variants were rare and almost never reported in the CEU population [57]. Therefore, among all of the SNPs examined from the ASVD risk interval, rs7341786 and rs7341791 appear most likely to influence *ANRIL* splicing. These SNPs are also in very strong linkage disequilibrium with the ASVD-associated SNP, rs10757278 (r^2^>0.96, based upon the nearest database SNPs in CEU), and therefore would be expected to correlate with *ANRIL* and *INK4/ARF* expression. However, it is important to note that other classes of germline variants (*e.g.* complex indels or alterations in repetitive elements) would not have been identified by our sequencing strategy, and therefore we are unable to exclude a role for other such variants in *ANRIL* expression and/or splicing.

## Discussion

Motivated by the role of other long, non-coding RNAs in PcG repression, we investigated whether *ANRIL* transcription and/or structure was SNP-dependent. In primary and cultured cell lines, we used RACE and RNA-seq to identify novel *ANRIL* variants (Figure 1 and Figure 2). Intriguingly, the central *ANRIL* exons were underrepresented in these data (Figure 1B and Figure 2B). This observation, along with non-colinear products detected by RACE, led to the unexpected discovery of multiple circular RNAs emanating from the *ANRIL* locus. Sequencing of these circular species showed non-sequential linkages between various *ANRIL* exons, especially those from the central portion of the transcript (*e.g.* exons 4-14) (Figure 4B and 4C). Expression of both circular and linear *ANRIL* isoforms proximal to the *INK4/ARF* locus strongly correlated with *INK4/ARF* transcription and the ASVD risk genotype at rs10757278 (Figure 5 and Figure S6). In contrast, distal *ANRIL* variants containing exons 18 and 19 were expressed in a genotype-independent manner and did not correlate with the levels of any *INK4/ARF* transcript. Using next-generation sequencing to genotype captured DNA from the ASVD risk interval, we identified a common pair of linked SNPs near exon 15 predicted to influence *ANRIL* splicing (Figure 6 and Table S1). Together, these findings suggest that common polymorphisms in the ASVD risk interval could modulate *ANRIL* transcription and/or splicing, thereby influencing PcG-mediated *INK4/ARF* repression and atherosclerosis susceptibility.

### *ANRIL* encodes multiple, non-abundant linear and circular species

Multiple *ANRIL* isoforms have been reported in the literature and EST databases, with some exhibiting differential expression patterns and SNP associations (Figure S2 and [28], [29]). Using RACE, RNA-seq and sensitive quantitative real-time PCR techniques, we now show that no single *ANRIL* species predominates *in vivo*, and that splicing to *MTAP* does not occur in cells with an intact 9p21 locus. Moreover, our analyses identified new *ANRIL* exons and variants, uncovering a novel group of circular *ANRIL* (*cANRIL*) species (Figure 4). Using independent approaches, we found that all *ANRIL* exons were expressed at very low levels, orders of magnitude lower than even the relatively rare *INK4/ARF* tumor suppressors, *p15^INK4b^* and *p16^INK4a^* (Figure 1B, Figure 2B, and Figure S4). This low level of expression is comparable to what we observed for other regulatory non-coding RNAs (*i.e. HOTAIR* and *Kcnq1ot1*) associated with PcG-mediated repression.

The discovery of non-colinear *ANRIL* species whose expression correlated with *INK4/ARF* transcription suggested that alternative splicing events might modify *ANRIL* structure leading to changes in PcG-mediated *INK4/ARF* repression. There are two major mechanisms by which non-colinear RNAs are thought to arise: *trans-*splicing and exon skipping [58]. During *trans-*splicing, a Y-branched RNA structure is formed that is sensitive to RNase R digestion [50]. In contrast, exon skipping events generate large lariat structures, which can then undergo *cis*-splicing to create RNase R-resistant circular RNAs. To confirm the circular nature of the *ANRIL* species, we showed that transcripts containing *ANRIL4-6* and *14-5* were resistant to RNase R degradation, were not polyadenylated and could be PCR amplified using sets of outward facing primers (Figure 3 and Figure 4). Therefore, *cANRIL* species appear to result from exon skipping events occurring during RNA splicing.

### *INK4/ARF* regulation and ASVD genotype

**Figure 7:**
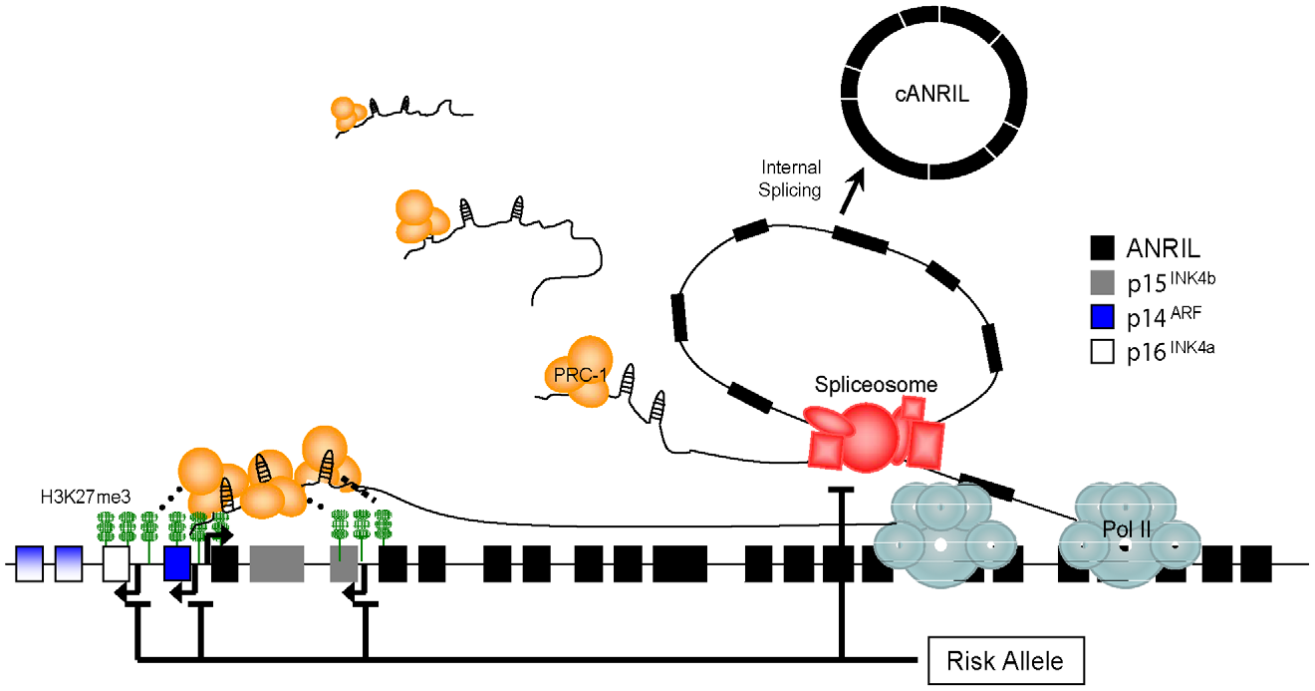
Model showing how 9p21 polymorphisms influence *ANRIL* isoform production to modulate *INK4/ARF* gene transcription. PcG complexes (e.g. PRC-1) are targeted to the coding INK4/ARF locus by *ANRIL*, and modulate its repression. Nascent *ANRIL* transcripts are spliced to produce circular ANRIL species (*cANRIL*). Causal variants in the ASVD risk interval modulate *ANRIL* transcription or splicing to influence *INK4/ARF* expression. See discussion for further description.

Based on these observations, we propose a model suggesting that common polymorphisms in 9p21.3 modifiy *INK4/ARF* gene expression through *cis*-regulation of *ANRIL* transcription and/or splicing (Figure 7). In turn, such changes in *ANRIL* levels or structure influence the ability of these ncRNAs to repress the *INK4/ARF* locus. Specifically, changes in PcG-mediated repression of *p15^INK4b^*, *p16^INK4a^* and/or *ARF* occur, altering potentially atherogenic cellular proliferation and ASVD risk as previously suggested [16]–[20], [26].

Prior studies provide evidence for this model. For example, we and others have described an effect of ASVD-genotype on *ANRIL* transcription [16], [30], [59], [60]. Moreover, meticulous allele-specific expression analyses by Cunnington *et al.* suggested that the *cis*-effects of 9p21 SNPs were stronger for *ANRIL* than for the coding *INK4/ARF* transcripts (20% vs. <8%)[60]. In contrast, the effect of these SNPs on expression of the coding *INK4/ARF* transcripts predominantly occurred in *trans*-. Such findings are consistent with our model whereby causal variants directly influence *ANRIL* structure in *cis*-, thereby controlling repression of the *INK4/ARF* locus, possibly in *trans*- (Figure 7). Likewise, recent work has supported a role for ncRNAs in *INK4/ARF* repression. Knockdown of the RNA helicase, Mov10, led to *INK4/ARF* deregulation, suggesting a role for RNA metabolism in regulation of the locus [61]. Supporting these data, Yap *et al*. recently demonstrated that the 5′ end of *ANRIL* contains stem-loop structures capable of binding CBX7, a member of the PRC-1 complex [62]. Disruption of this interaction led to premature senescence marked by increased *INK4/ARF* expression. Therefore, strong evidence supports the notion that *ANRIL* expression correlates with ASVD-SNP genotype and that PcG-mediated repression of the *INK4/ARF* locus is modulated by this expression.

The outstanding question of this model is the biochemical mechanism whereby causal variants at 9p21.3 located more than 100 kb distant from the proximal exons of *ANRIL* (Figure S1) modulate *ANRIL* expression and/or structure. Our data are consistent with a “transcriptional model” wherein distal *cis*-regulatory elements within the ASVD risk interval directly influence *ANRIL* transcription. Jarinova *et al.* have identified an enhancer sequence within *ANRIL* intron 17, which in heterologous reporter assays, enhanced transcription in a genotype-specific manner [30]. Consistent with this view, we also observed a significant effect of ASVD-SNP genotype on the expression of the linear *ANRIL1-2* transcript (Figure 5B). As such, the correlation of *cANRIL* expression with ASVD-SNP genotype would presumably reflect passive production of these circular species in the setting of increased or decreased in total *ANRIL* transcription. However, this model would not easily explain the positive correlation we observed between the expression of coding *INK4/ARF* transcripts and proximal *ANRIL* exons (Figure 5A and Figure S6), as the 5′end of *ANRIL* is reported to foster *INK4/ARF* repression [62].

Alternatively, we believe our present data are more consistent with a “splicing model” in which causal variants in the ASVD risk interval influence *ANRIL* splicing by regulating exon skipping. Provocatively, the ASVD risk interval includes exon 15 where the termination of most exon skipping events that produce *cANRIL* occur (Figure 4B and 4C). Using next-generation sequencing of this region in individuals biased to harbor causal variants, we identified exon 15 SNPs (rs7341786 and rs7341791) which are in strong linkage disequilibrium with the ASVD-associated SNP rs10757278 (r^2^>0.96; Table S1). These polymorphisms have high minor allele frequencies in our sample and the CEU population and are predicted to alter *ANRIL* splicing (Figure 6, Table S1). In particular, the major allele (‘A’) of rs7341786, detected only in individuals homozygous for the A-allele at rs10757278, is predicted to increase the strength of exon 15 as a splice acceptor, which would promote the expression of spliced RNA circles ending in exon 14, as observed (Figure 4B and 4C).

For the splicing model to explain the observed positive correlation between the expression of *ANRIL* and the coding *INK4/ARF* transcripts, distinct species of *ANRIL* would need to differ in their ability to repress the *INK4/ARF* locus. Supporting this possibility, PcG-mediated repression of the murine *Kcnq1* locus is dependent upon the length of the long, ncRNA, *Kcnq1ot1* [63], [64]. Specifically, longer *Kcnq1ot1* transcripts are associated with increased PcG recruitment and Kcnq1 silencing potential. Therefore, shorter *ANRIL* variants (*i.e.* those lacking exons 4–16) generated by exon skipping events may also be less efficient at repressing the *INK4/ARF* locus. Important predictions of the splicing model are that *cANRIL* expression (reflecting *ANRIL* splicing) should correlate with *INK4/ARF* expression (which it does, Figure 5A and Figure S6), that individuals homozygous for the ‘A’ allele of rs7341786 (and rs10757278) should exhibit increased production of *cANRIL* species containing exon 14 but not exon 15 (which they do, see Figure 4B and 4C) and that these individuals with increased propensity for splicing should exhibit de-repressed *INK4/ARF* expression (which they do, [16]).

A caveat to the splicing model is that the identified exon 15 SNPs need not be the true causal variant(s) influencing *ANRIL* splicing. It is possible that other polymorphisms not detected by our sequencing strategy could regulate *ANRIL* splicing. In particular, the sequencing strategy employed would not find differences between the pooled samples in repetitive regions, such as the large *LINE* element in the 12^th^ intron of *ANRIL* (Figure 6A). *ANRIL* harbors several *LINE* and *SINE* elements, and such repetitive motifs have been reported to modulate RNA splicing in other systems [65], [66]. Therefore, while the exon 15 SNPs appear to be prime candidates to regulate *ANRIL* splicing, a variety of other classes of polymorphisms could also influence splicing and would not have been observed by the chosen sequencing approach.

Importantly, the splicing and transcriptional models are not mutually exclusive. A single causal variant may influence both processes or there may be multiple causal variants that influence either process within the ASVD risk interval. Finally, while we believe the correlation of *cANRIL* expression and ASVD-SNP genotype is most likely explained by an effect of common 9p21 polymorphisms on transcription and/or splicing, a third possibility also exists. While circular RNA byproducts of exon skipping have generally been regarded as inconsequential, circular RNAs with catalytic activities (*e.g.* group I and some group II introns) are well described in bacteria, lower eukaryotes, plants [67]. In addition, some viroids and the hepatitis delta satellite virus have circular RNA genomes [68], [69]. Although we are not aware of any endogenously produced circular RNA with discrete function in mammals, clearly circular RNAs species can possess independent functions in non-mammalian species, and we remain open to the possibility that *cANRIL* itself can directly participate in *INK4/ARF* regulation.

In summary, this work links ASVD-genotype to *ANRIL* structure and *INK4/ARF* regulation, providing evidence for what we believe is a first association between endogenous circular RNA expression and a mammalian phenotype (ASVD). Even were *cANRIL* expression merely a marker of exon skipping with no specific biologic function, we believe its expression may be a useful marker of *ANRIL* isoform selection, which our findings suggest is of pathogenic relevance to ASVD susceptibility. We believe this work has implications beyond ASVD, as altered *ANRIL* splicing could influence *INK4/ARF* expression, explaining the association of other nearby 9p21 SNPs with a variety of non-ASVD phenotypes in humans including longevity, type II diabetes, endometriosis and several tumors types [70]–[77].

## Materials and Methods

### Ethics statement

Research involving human subjects was approved by the University of North Carolina Institutional Review Board and all participants provided informed, written consent.

### Cell lines and culture conditions

WM266-4, UACC 257, A2058, A375, SUM-149, RPMI-8322 and telomerized Hs68 cells were obtained and grown as previously described [78]–[80]. MDA-MB-468, MDA-MB-436, MDA-MB-231, MCF7, BT-474, BT-549, T-47D, COLO 205, T84, LoVo, LS 174T, SW480, LS1034, HeLa, HUVEC, IMR90, Ramos, Raji, Jurkat and U-87 cells were originally obtained from ATCC and cultured as suggested.

### 3′ and 5′ rapid amplification of cDNA ends (RACE)

CD3 positive T-cells were isolated from human peripheral blood samples as previously described [42]. RNA was generated from proliferating cell lines and isolated human T-cells using the RNAeasy system (Qiagen Inc., Valencia, CA). 3′ and 5′ RACE was performed as described in the Firstchoice RLM-RACE manual (Ambion Inc., Austin, TX). This procedure is optimized for the detection of rare transcripts and provides additional steps to improve the specificity of mRNA amplification. Gene-specific primers were designed within *ANRIL* exons 4 and 6 as shown in Figure 1A and Table S2; RACE primers for other *ANRIL* exons tested did not amplify chromosome 9 specific products. All PCR reactions were conducted using SuperTaq-Plus (Ambion) in a Bio-Rad DNA Engine thermocycler. SuperTaq-Plus is a high fidelity, long range polymerase with the capability to amplify complex DNAs such as repetitive *SINE*, *LINE* and *Alu* elements. Cycling conditions for 5′RACE were: 94°C 3 min, 34×[94°C 30 s, 60°C 30 s, 68°C 3 min], 68°C 5 min (inner reaction) and 94°C 3 min, 34×[94°C 30 s, 62°C 30 s, 68°C 3 min], 68°C 5 min (outer reaction). Cycling conditions for 3′RACE were: 94°C 3 min, 34× [94°C 30 s, 57 or 60°C 30 s, 68°C 3 min] (outer reaction) and 94°C 3 min, 34×[94°C 30 s, 60°C 30 s, 68°C 3 min] (inner reaction). Cloning of the resulting PCR products was conducted using the TOPO-Blunt cloning kit (Invitrogen). Sequencing of the resulting clones was conducted using both M13F and M13R primers.

### Quantitative real-time PCR (qRT-PCR)

RNA was isolated using the Qiagen RNAeasy Kit and subjected to reverse transcription using the ImPromII reverse transcription system (Promega, Madison, WI). RNA samples from breast and colorectal cell lines were kindly provided by Drs. N. Mitin and J. Yeh (UNC). Taqman primer and probe sets for the detection of *ANRIL* exons 1–2, 4–6, 14–5, and 18–19 as well as those for *p15^INK4b^*, *p16^INK4a^*, *ARF* and 18S rRNA are described in Table S2. The *ANRIL* primer sets were designed to span at least one intron and were shown to have high specificity with linear amplification efficiencies between 88 and 94% (Figure S3). Final primer and probe concentrations were 900 and 250 nM, respectively. Products from the *ANRIL* 1–2, 4–6, 14–5 and 18–19 qRT-PCR reactions were cloned separately into the pBluntII-TOPO vector (Invitrogen) and verified. Real-time PCR was carried out in triplicate on an ABI 7900HT thermocyler.

For relative expression studies, differentials were first calculated between each sample and the average 18S cycle threshold (Ct) for the entire experiment. Transcript Ct values were normalized using the equation: Expression  =  Lower limit of detection (37 or 40) - (Ct _target_ – Ct_ 18S differential_) and plotted on a log_2_ scale. The relative expression of *p16^INK4a^*, *p15^INK4b^*, *ARF* and *ANRIL4-6* in PBTLs has been previously reported [42]; in this work, these same samples were reanalyzed for *ANRIL1-2*, *ANRIL14-5* and *ANRIL18-19* expression as shown in Figure 5 and Figure S6. In Figure 5 and Figure S6, data was corrected to account for batch effects between the pilot and verification datasets previously described [42]. To determine the absolute number of transcripts present, a standard curve of five dilutions was generated for each experimental plate using known amounts of linearized plasmid containing the target sequence. The number of molecules detected in each sample was calculated using the equation: #Molecules  =  10∧((Ct – y-intercept)/slope). Primer efficiencies were calculated using the equation: Efficiency  =  (10∧(-1/slope))-1. Multiple comparison plots and statistical analysis appearing in Figure 5A and Figure S6 was performed using the gpairs function of the R YaleToolkit library.

### Analysis of RNA-seq data

A total of 368,846,235 reads generated on the Illumina platform (study SRP002274) were downloaded from the NCBI Short Read Archive. The reads were first screened for unique 20mers deriving from chromosome 9∶21,700,000–22,300,000 using the UCSC genome bowser Duke uniqueness mapability table. The resulting reads were mapped using the TopHat spliced aligner (PMID: 19289445) to the reference human genome (hg18). The resulting coverage plot was imported into the UCSC genome browser for display. Also analyzed were two independent CalTech ENCODE mRNA-seq datasets (http://genome.ucsc.edu/cgi-bin/hgTrackUi?g=wgEncodeCaltechRnaSeq) from HeLa cells. These datasets were chosen because they lacked deletion of 9p21 and showed the highest level of *ANRIL* expression of any publically available RNA-seq data we analyzed. For Figure 2B, the peak number of reads at a given base (SRP002274 data) or peak reads per kilobase mapping (CalTech HeLa data) were quantified and scaled relative to the average exonic coverage.

### RNase R digestion

Total RNA was isolated from proliferating Hs68 and IMR90 cells using a Qiagen RNAeasy kit. Equal amounts of RNA (43–50 µg, depending on the experiment) were incubated with or without 40 U of Rnase R (RNR07250, Epicentre Biotechnologies) for 2.5 hours at 37°C. The resulting RNA was purified using the RNAeasy column and quantified. Equal amounts of RNA were then subjected to reverse transcription using the ImPromII reverse transcription system and a mixture of random hexamer and oligo dT primers (Promega, Madison, WI). Transcripts were quantified using Taqman-based real-time PCR.

### Loop PCR

PCR primers pointing in opposite directions on *ANRIL* exons 1, 4, 6, 13, 16 and 18 were designed using Primer3 software and analyzed for hairpins using Netprimer (Premier Biosoft and http://frodo.wi.mit.edu/primer3/) (Table S2). PCR reactions were conducted using cDNA representing 15 ng of mock or Rnase R-treated RNA. Reactions were performed using Apex Hot Start Taq DNA polymerase and Buffer 2 (Genesee Scientific) in a Bio-Rad DNA Engine thermocycler. The cycling conditions were as follows: 95°C 15 min, 40×[94°C 30 s, 59°C 30 s, 72°C 1 min], 72°C 2 min. The resulting PCR products were cloned into the TOPO-Blunt cloning kit (Invitrogen) and sequenced using M13F and M13R primers.

### Sequence capture and next-generation sequencing

Genomic DNA was generated from T-cells of healthy human volunteers of known rs10757278 genotype, age and *p16^INK4a^* expression status using the Qiagen Peripheral Blood DNA isolation kit. For sequence capture, 21 ug of DNA was pooled from five individuals homozygous for the G-allele of rs10757278 and another five individuals homozygous for the A-allele. Samples were sent to NimbleGen for sequence capture using a tiled array spanning human chromosome 9 (22,054,888-22,134,171). The resulting amplified DNA fragments were analyzed at the UNC Genome Analysis Facility using both Illumina GAII and Roche 454 technology. For sequencing on the Illumina platform, DNA was randomly sheered and appropriate adapters ligated. Resulting sequences were aligned to the entire human genome (hg18) using MAQ, SOAP, and gsMapper software [52], [53]. MAQ was run with default settings and output was translated into BAM format. SOAP alignment was performed allowing up to 10 gap bases and 2 mismatches and also translated into BAM format. Mapping with gsMapper was performed with default settings. SNP calls from MAQ and SOAP were generated using the pileup function of the SAMtools library [81]. Calls were culled to include only those SNPs appearing in ≥20 percent of reads.

## Supporting Information

Figure S1 Polymorphisms within the *INK4/ARF* locus linked to age-related diseases. A, Schematic diagram of the 9p21 locus depicting the *INK4/ARF* tumor suppressors, *ANRIL* and the ASVD risk interval. The captured (“tiling”) region for next generation DNA sequencing is indicated. B, The localization of SNPs linked in the literature to age-related diseases including is shown. T2D- type II diabetes, CAD- coronary artery disease, MI-myocardial infarction, AD-Alzheimer's disease C, A heatmap depicting the SNP linkage disequilibrium (D′/LOD) was generated from the Hapmap CEU population using Haploview software. The strength of linkage disequilibrium increases from white to blue to red: white (disequilibrium coefficient (D′)<1 and LOD score <2); blue (D′ = 1 and LOD score <2); pink (D′<1 and LOD score ≥2); and red (D′ = 1 and LOD score ≥2). The heatmap can be aligned to the depicted 9p21 image as shown in the diagram above.
(1.37 MB TIF)

Figure S2 Schematic of previously reported *ANRIL* variants. All Ensembl (blue) and GenBank (black) records for *ANRIL* (*CDKN2BAS*) are shown. Some sequences are derived from cDNA sequencing whereas others were inferred by EST assembly.
(0.64 MB TIF)

Figure S3 Efficiency curves for Taqman probe strategies. Cloned cDNAs or PCR products corresponding to the Taqman target sequences were linearized and quantified. For each realtime PCR run, a standard curve of 5 independent dilutions was run in triplicate. Primer efficiency was calculated using the formula: Efficiency  = 10∧(-1/slope)-1. Shown are representative graphs from individual experiments.
(0.52 MB TIF)

Figure S4 Expression of 9p21 transcripts in transformed and non-transformed cell lines. As described in Figure 1B, RNA was harvested, reverse transcribed and quantitative real-time PCR performed. Bars represent the log10 of the average number of molecules detected. The error bars denote the standard deviation between three replicates. The letter ‘D’ denotes deletion events previously reported in the literature. ‘M’ indicates gene methylation. Breast cancer are shown in pink, colorectal cancers in blue, melanomas in black, hematological malignancies in green, cervical carcinomas in turquoise, glioblastomas in gold and nontransformed cells in red.
(0.58 MB TIF)

Figure S5 *ANRIL14-5* expression is detected in a wide variety of cell types. cDNA generated as described in Figure 1B was assayed for *ANRIL14-5* expression using the Taqman strategy shown in Figure 3A. Bars represent the log10 of the average number of molecules detected. The error bars denote the standard deviation between three replicates. Cell lines are color coded as in Figure S4.
(0.50 MB TIF)

Figure S6 Correlation of *ANRIL* and *INK4/ARF* expression in primary peripheral blood T-lymphocytes. Diagram depicting the correlations between 9p21 transcripts in primary peripheral blood T-lymphocytes from 106 patients. Taqman anlaysis of *ANRIL* and *INK4/ARF* transcripts was conducted and normalized as described in Materials and Methods. Data for *ANRIL4-6*, *p16^INK4a^*, *p15^INK4b^* and *ARF* expression were previously reported [42]. Scatter plots, below the diagonal, show the relationships between all pairs of transcripts on a log2 scale. Linear regression is depicted in red. Boxes above the diagonal list and are color coded by r-value. A star (*) indicates significant associations (p<0.05). Histograms along the diagonal show the distribution of expression for each transcript assayed. Due to limitations in sample availability, *ANRIL 1-2* and *ANRIL 14-5* levels were not determined for several individuals as indicated (n = 94 and 98, respectively).
(1.55 MB TIF)

Table S1 Splice site analysis of polymorphisms in the ASVD risk interval near *ANRIL* exon-intron boundaries. SNPs within 200 bp of an *ANRIL* inton-exon boundary were analyzed for their effects on putative exon splicing enhancer (ESE), exon splicing silencer (ESS), intron splicing enhancer (ISE), and intron splicing silencer (ISS) sequences as described ([54], [55] and Z. Wang unpublished data). A score of -1 indicates that the minor allele destroys one *cis-*element and +1 indicates that the minor allele creates one *cis*-element. SNPs identified as unique to the AA or GG samples using sequence capture are shown in blue. Those identified in the HapMap database are depicted in black. The position of each intronic SNP relative to the nearest *ANRIL* exon is given under the ‘*ANRIL* Position’ column. Exonic SNPs in this column, list the exon in which they occur. If available, the minor allele frequency from Utah residents with ancestry from northern and western Europe (HapMap3, CEU) is given in the ‘CEU Minor Allele’ column. H- Hapmap3; GG- individuals homozygous for the ‘G’ allele at rs10757278.
(0.59 MB TIF)

Table S2 Primers used for RACE, Taqman, and PCR analysis.
(0.67 MB TIF)

## References

[1] World Health Organization W (2009). Cardiovascular diseases Fact Sheet.

[2] Biros E, Cooper M, Palmer LJ, Walker PJ, Norman PE (2010). Association of an allele on chromosome 9 and abdominal aortic aneurysm.. Atherosclerosis.

[3] Bown MJ, Braund PS, Thompson J, London NJ, Samani NJ (2008). Association between the coronary artery disease risk locus on chromosome 9p21.3 and abdominal aortic aneurysm.. Circ Cardiovasc Genet.

[4] Thompson AR, Golledge J, Cooper JA, Hafez H, Norman PE (2009). Sequence variant on 9p21 is associated with the presence of abdominal aortic aneurysm disease but does not have an impact on aneurysmal expansion.. Eur J Hum Genet.

[5] Helgadottir A, Thorleifsson G, Manolescu A, Gretarsdottir S, Blondal T (2007). A common variant on chromosome 9p21 affects the risk of myocardial infarction.. Science.

[6] Helgadottir A, Thorleifsson G, Magnusson KP, Gretarsdottir S, Steinthorsdottir V (2008). The same sequence variant on 9p21 associates with myocardial infarction, abdominal aortic aneurysm and intracranial aneurysm.. Nat Genet.

[7] McPherson R, Pertsemlidis A, Kavaslar N, Stewart A, Roberts R (2007). A common allele on chromosome 9 associated with coronary heart disease.. Science.

[8] Matarin M, Brown WM, Singleton A, Hardy JA, Meschia JF (2008). Whole genome analyses suggest ischemic stroke and heart disease share an association with polymorphisms on chromosome 9p21.. Stroke.

[9] Gschwendtner A, Bevan S, Cole JW, Plourde A, Matarin M (2009). Sequence variants on chromosome 9p21.3 confer risk for atherosclerotic stroke.. Ann Neurol.

[10] Smith JG, Melander O, Lovkvist H, Hedblad B, Engstrom G (2009). Common genetic variants on chromosome 9p21 confers risk of ischemic stroke: a large-scale genetic association study.. Circ Cardiovasc Genet.

[11] Cluett C, McDermott MM, Guralnik J, Ferrucci L, Bandinelli S (2009). The 9p21 myocardial infarction risk allele increases risk of peripheral artery disease in older people.. Circ Cardiovasc Genet.

[12] Ye S, Willeit J, Kronenberg F, Xu Q, Kiechl S (2008). Association of genetic variation on chromosome 9p21 with susceptibility and progression of atherosclerosis: a population-based, prospective study.. J Am Coll Cardiol.

[13] Broadbent HM, Peden JF, Lorkowski S, Goel A, Ongen H (2008). Susceptibility to coronary artery disease and diabetes is encoded by distinct, tightly linked SNPs in the ANRIL locus on chromosome 9p.. Hum Mol Genet.

[14] Seidelmann SB, Kuo C, Pleskac N, Molina J, Sayers S (2008). Athsq1 is an atherosclerosis modifier locus with dramatic effects on lesion area and prominent accumulation of versican.. Arterioscler Thromb Vasc Biol.

[15] Kim WY, Sharpless NE (2006). The regulation of INK4/ARF in cancer and aging.. Cell.

[16] Liu Y, Sanoff HK, Cho H, Burd CE, Torrice C (2009). INK4/ARF transcript expression is associated with chromosome 9p21 variants linked to atherosclerosis.. PLoS One.

[17] Wessely R (2010). Atherosclerosis and cell cycle: put the brakes on! Critical role for cyclin-dependent kinase inhibitors.. J Am Coll Cardiol.

[18] Boehm M, Nabel EG (2003). The cell cycle and cardiovascular diseases.. Prog Cell Cycle Res.

[19] Gizard F, Amant C, Barbier O, Bellosta S, Robillard R (2005). PPAR alpha inhibits vascular smooth muscle cell proliferation underlying intimal hyperplasia by inducing the tumor suppressor p16INK4a.. J Clin Invest.

[20] Gonzalez-Navarro H, Abu Nabah YN, Vinue A, Andres-Manzano MJ, Collado M (2010). p19(ARF) deficiency reduces macrophage and vascular smooth muscle cell apoptosis and aggravates atherosclerosis.. J Am Coll Cardiol.

[21] Kalinina N, Agrotis A, Antropova Y, Ilyinskaya O, Smirnov V (2004). Smad expression in human atherosclerotic lesions: evidence for impaired TGF-beta/Smad signaling in smooth muscle cells of fibrofatty lesions.. Arterioscler Thromb Vasc Biol.

[22] Reynisdottir I, Polyak K, Iavarone A, Massague J (1995). Kip/Cip and Ink4 Cdk inhibitors cooperate to induce cell cycle arrest in response to TGF-beta.. Genes Dev.

[23] Grainger DJ (2004). Transforming growth factor beta and atherosclerosis: so far, so good for the protective cytokine hypothesis.. Arterioscler Thromb Vasc Biol.

[24] Janzen V, Forkert R, Fleming HE, Saito Y, Waring MT (2006). Stem-cell ageing modified by the cyclin-dependent kinase inhibitor p16INK4a.. Nature.

[25] Yvan-Charvet L, Pagler T, Gautier EL, Avagyan S, Siry RL (2010). ATP-binding cassette transporters and HDL suppress hematopoietic stem cell proliferation.. Science.

[26] Visel A, Zhu Y, May D, Afzal V, Gong E (2010). Targeted deletion of the 9p21 non-coding coronary artery disease risk interval in mice.. Nature.

[27] Pasmant E, Laurendeau I, Heron D, Vidaud M, Vidaud D (2007). Characterization of a germ-line deletion, including the entire INK4/ARF locus, in a melanoma-neural system tumor family: identification of ANRIL, an antisense noncoding RNA whose expression coclusters with ARF.. Cancer Res.

[28] Folkersen L, Kyriakou T, Goel A, Peden J, Malarstig A (2009). Relationship between CAD risk genotype in the chromosome 9p21 locus and gene expression. Identification of eight new ANRIL splice variants.. PLoS One.

[29] Kyriakou T, Pal A, Peden J, Green F, Gloyn A (2009). ANRIL, The non-coding RNA present in the chromosome 9 CAD associated locus, has multiple splice variants and a potential regulatory role in CDKN2B expression.. Atherosclerosis.

[30] Jarinova O, Stewart AF, Roberts R, Wells G, Lau P (2009). Functional analysis of the chromosome 9p21.3 coronary artery disease risk locus.. Arterioscler Thromb Vasc Biol.

[31] Jacobs JJ, Kieboom K, Marino S, DePinho RA, van Lohuizen M (1999). The oncogene and Polycomb-group gene bmi-1 regulates cell proliferation and senescence through the ink4a locus.. Nature.

[32] Dhawan S, Tschen SI, Bhushan A (2009). Bmi-1 regulates the Ink4a/Arf locus to control pancreatic beta-cell proliferation.. Genes Dev.

[33] Chen H, Gu X, Su IH, Bottino R, Contreras JL (2009). Polycomb protein Ezh2 regulates pancreatic beta-cell Ink4a/Arf expression and regeneration in diabetes mellitus.. Genes Dev.

[34] Kotake Y, Cao R, Viatour P, Sage J, Zhang Y (2007). pRB family proteins are required for H3K27 trimethylation and Polycomb repression complexes binding to and silencing p16INK4alpha tumor suppressor gene.. Genes Dev.

[35] Bracken AP, Kleine-Kohlbrecher D, Dietrich N, Pasini D, Gargiulo G (2007). The Polycomb group proteins bind throughout the INK4A-ARF locus and are disassociated in senescent cells.. Genes Dev.

[36] Gil J, Peters G (2006). Regulation of the INK4b-ARF-INK4a tumour suppressor locus: all for one or one for all.. Nat Rev Mol Cell Biol.

[37] Terranova R, Yokobayashi S, Stadler MB, Otte AP, van Lohuizen M (2008). Polycomb group proteins Ezh2 and Rnf2 direct genomic contraction and imprinted repression in early mouse embryos.. Dev Cell.

[38] Rinn JL, Kertesz M, Wang JK, Squazzo SL, Xu X (2007). Functional demarcation of active and silent chromatin domains in human HOX loci by noncoding RNAs.. Cell.

[39] Zhao J, Sun BK, Erwin JA, Song JJ, Lee JT (2008). Polycomb proteins targeted by a short repeat RNA to the mouse X chromosome.. Science.

[40] Pandey RR, Mondal T, Mohammad F, Enroth S, Redrup L (2008). Kcnq1ot1 antisense noncoding RNA mediates lineage-specific transcriptional silencing through chromatin-level regulation.. Mol Cell.

[41] Gupta RA, Shah N, Wang KC, Kim J, Horlings HM (2010). Long non-coding RNA HOTAIR reprograms chromatin state to promote cancer metastasis.. Nature.

[42] Liu Y, Sanoff HK, Cho H, Burd CE, Torrice C (2009). Expression of p16(INK4a) in peripheral blood T-cells is a biomarker of human aging.. Aging Cell.

[43] Hara E, Smith R, Parry D, Tahara H, Stone S (1996). Regulation of p16CDKN2 expression and its implications for cell immortalization and senescence.. Mol Cell Biol.

[44] Krishnamurthy J, Torrice C, Ramsey MR, Kovalev GI, Al-Regaiey K (2004). Ink4a/Arf expression is a biomarker of aging.. J Clin Invest.

[45] Au KF, Jiang H, Lin L, Xing Y, Wong WH (2010). Detection of splice junctions from paired-end RNA-seq data by SpliceMap.. Nucleic Acids Res.

[46] Nielsen GP, Stemmer-Rachamimov AO, Shaw J, Roy JE, Koh J (1999). Immunohistochemical survey of p16INK4A expression in normal human adult and infant tissues.. Lab Invest.

[47] Schmid M, Sen M, Rosenbach MD, Carrera CJ, Friedman H (2000). A methylthioadenosine phosphorylase (MTAP) fusion transcript identifies a new gene on chromosome 9p21 that is frequently deleted in cancer.. Oncogene.

[48] Chen K, Wallis JW, McLellan MD, Larson DE, Kalicki JM (2009). BreakDancer: an algorithm for high-resolution mapping of genomic structural variation.. Nat Methods.

[49] Zaphiropoulos PG (1996). Circular RNAs from transcripts of the rat cytochrome P450 2C24 gene: correlation with exon skipping.. Proc Natl Acad Sci U S A.

[50] Suzuki H, Zuo Y, Wang J, Zhang MQ, Malhotra A (2006). Characterization of RNase R-digested cellular RNA source that consists of lariat and circular RNAs from pre-mRNA splicing.. Nucleic Acids Res.

[51] Dixon RJ, Eperon IC, Hall L, Samani NJ (2005). A genome-wide survey demonstrates widespread non-linear mRNA in expressed sequences from multiple species.. Nucleic Acids Res.

[52] Li H, Durbin R (2009). Fast and accurate short read alignment with Burrows-Wheeler transform.. Bioinformatics.

[53] Li R, Li Y, Kristiansen K, Wang J (2008). SOAP: short oligonucleotide alignment program.. Bioinformatics.

[54] Wang Z, Rolish ME, Yeo G, Tung V, Mawson M (2004). Systematic identification and analysis of exonic splicing silencers.. Cell.

[55] Fairbrother WG, Yeh RF, Sharp PA, Burge CB (2002). Predictive identification of exonic splicing enhancers in human genes.. Science.

[56] Wang Z, Burge CB (2008). Splicing regulation: from a parts list of regulatory elements to an integrated splicing code.. Rna.

[57] Frazer KA, Ballinger DG, Cox DR, Hinds DA, Stuve LL (2007). A second generation human haplotype map of over 3.1 million SNPs.. Nature.

[58] Nigro JM, Cho KR, Fearon ER, Kern SE, Ruppert JM (1991). Scrambled exons.. Cell.

[59] Holdt LM, Beutner F, Scholz M, Gielen S, Gabel G (2010). ANRIL expression is associated with atherosclerosis risk at chromosome 9p21.. Arterioscler Thromb Vasc Biol.

[60] Cunnington MS, Santibanez Koref M, Mayosi BM, Burn J, Keavney B (2010). Chromosome 9p21 SNPs Associated with Multiple Disease Phenotypes Correlate with ANRIL Expression.. PLoS Genet.

[61] Messaoudi-Aubert SE, Nicholls J, Maertens GN, Brookes S, Bernstein E (2010). Role for the MOV10 RNA helicase in polycomb-mediated repression of the INK4a tumor suppressor.. Nat Struct Mol Biol.

[62] Yap KL, Li S, Munoz-Cabello AM, Raguz S, Zeng L (2010). Molecular interplay of the noncoding RNA ANRIL and methylated histone H3 lysine 27 by polycomb CBX7 in transcriptional silencing of INK4a.. Mol Cell.

[63] Mancini-Dinardo D, Steele SJ, Levorse JM, Ingram RS, Tilghman SM (2006). Elongation of the Kcnq1ot1 transcript is required for genomic imprinting of neighboring genes.. Genes Dev.

[64] Kanduri C, Thakur N, Pandey RR (2006). The length of the transcript encoded from the Kcnq1ot1 antisense promoter determines the degree of silencing.. Embo J.

[65] Dixon RJ, Eperon IC, Samani NJ (2007). Complementary intron sequence motifs associated with human exon repetition: a role for intragenic, inter-transcript interactions in gene expression.. Bioinformatics.

[66] Cocquerelle C, Daubersies P, Majerus MA, Kerckaert JP, Bailleul B (1992). Splicing with inverted order of exons occurs proximal to large introns.. Embo J.

[67] Bonen L, Vogel J (2001). The ins and outs of group II introns.. Trends Genet.

[68] Kos A, Dijkema R, Arnberg AC, van der Meide PH, Schellekens H (1986). The hepatitis delta (delta) virus possesses a circular RNA.. Nature.

[69] Tsagris EM, Martinez de Alba AE, Gozmanova M, Kalantidis K (2008). Viroids.. Cell Microbiol.

[70] Zeggini E, Weedon MN, Lindgren CM, Frayling TM, Elliott KS (2007). Replication of genome-wide association signals in UK samples reveals risk loci for type 2 diabetes.. Science.

[71] Scott LJ, Mohlke KL, Bonnycastle LL, Willer CJ, Li Y (2007). A genome-wide association study of type 2 diabetes in Finns detects multiple susceptibility variants.. Science.

[72] Shete S, Hosking FJ, Robertson LB, Dobbins SE, Sanson M (2009). Genome-wide association study identifies five susceptibility loci for glioma.. Nat Genet.

[73] Wrensch M, Jenkins RB, Chang JS, Yeh RF, Xiao Y (2009). Variants in the CDKN2B and RTEL1 regions are associated with high-grade glioma susceptibility.. Nat Genet.

[74] Uno S, Zembutsu H, Hirasawa A, Takahashi A, Kubo M (2010). A genome-wide association study identifies genetic variants in the CDKN2BAS locus associated with endometriosis in Japanese.. Nat Genet.

[75] Bei JX, Li Y, Jia WH, Feng BJ, Zhou G (2010). A genome-wide association study of nasopharyngeal carcinoma identifies three new susceptibility loci.. Nat Genet.

[76] Emanuele E, Fontana JM, Minoretti P, Geroldi D (2010). Preliminary evidence of a genetic association between chromosome 9p21.3 and human longevity.. Rejuvenation Res.

[77] Sebastiani P, Solovieff N, Puca A, Hartley SW, Melista E (2010). Genetic Signatures of Exceptional Longevity in Humans.. Science.

[78] Hanker AB, Morita S, Repasky GA, Ross DT, Seitz RS (2008). Tools to study the function of the Ras-related, estrogen-regulated growth inhibitor in breast cancer.. Methods Enzymol.

[79] Brookes S, Rowe J, Ruas M, Llanos S, Clark PA (2002). INK4a-deficient human diploid fibroblasts are resistant to RAS-induced senescence.. Embo J.

[80] Shields JM, Thomas NE, Cregger M, Berger AJ, Leslie M (2007). Lack of extracellular signal-regulated kinase mitogen-activated protein kinase signaling shows a new type of melanoma.. Cancer Res.

[81] Li H, Handsaker B, Wysoker A, Fennell T, Ruan J (2009). The Sequence Alignment/Map format and SAMtools.. Bioinformatics.

