# Supplementary figures and images for: Expression of Linear and Novel Circular Forms of an INK4/ARF-Associated Non-Coding RNA Correlates with Atherosclerosis Risk
Source: PLoS Genet. 2010 Dec 2;6(12):e1001233. doi: 10.1371/journal.pgen.1001233 (PMC2996334; doi:10.1371/journal.pgen.1001233)

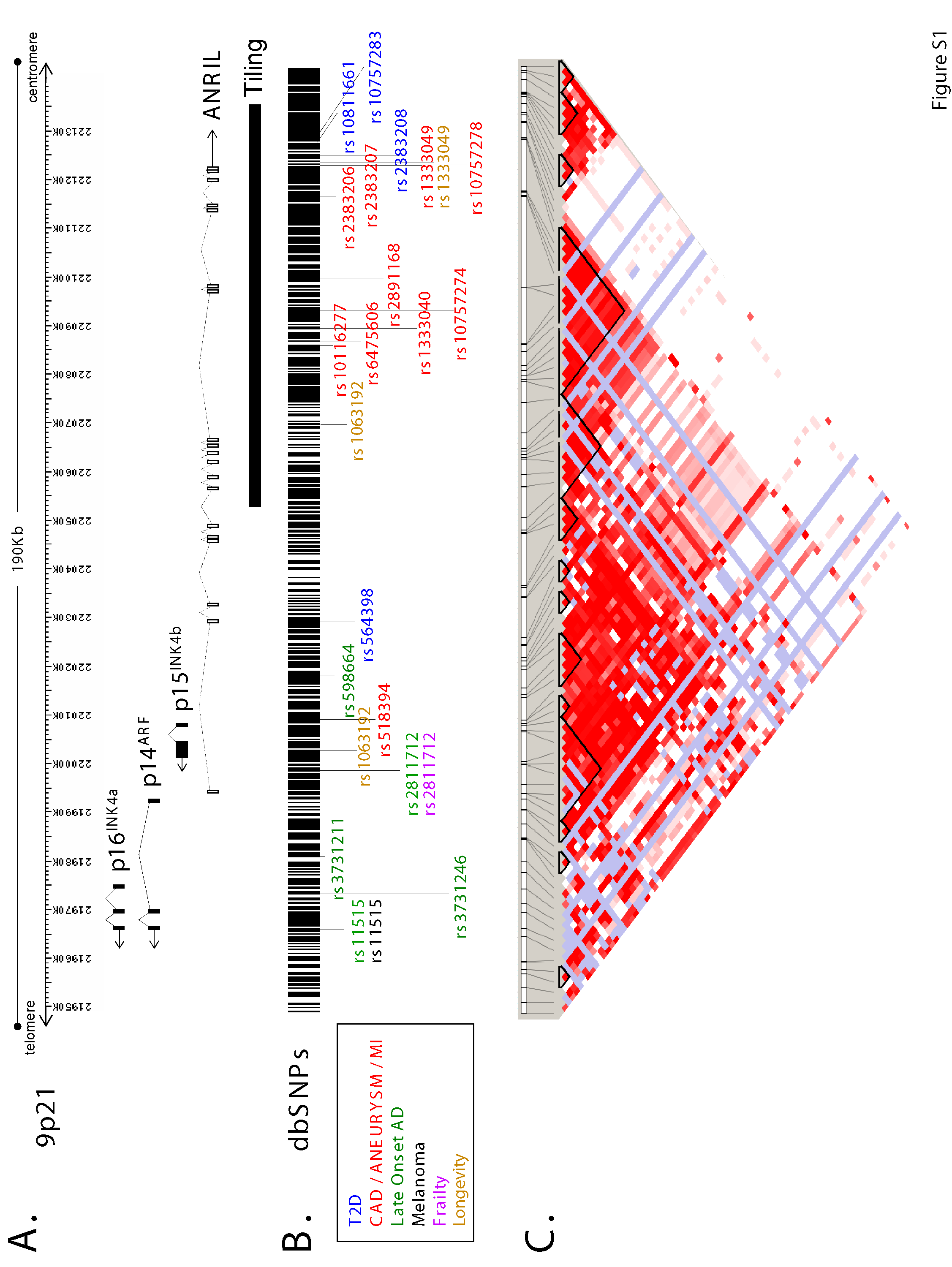

Supplement: Figure S1 — Polymorphisms within the INK4/ARF locus linked to age-related diseases. A, Schematic diagram of the 9p21 locus depicting the INK4/ARF tumor suppressors, ANRIL and the ASVD risk interval. The captured (“tiling”) region for next generation DNA sequencing is indicated. B, The localization of SNPs linked in the literature to age-related diseases including is shown. T2D- type II diabetes, CAD- coronary artery disease, MI-myocardial infarction, AD-Alzheimer's disease C, A heatmap depicting the SNP linkage disequilibrium (D′/LOD) was generated from the Hapmap CEU population using Haploview software. The strength of linkage disequilibrium increases from white to blue to red: white (disequilibrium coefficient (D′)<1 and LOD score <2); blue (D′ = 1 and LOD score <2); pink (D′<1 and LOD score ≥2); and red (D′ = 1 and LOD score ≥2). The heatmap can be aligned to the depicted 9p21 image as shown in the diagram above. (1.37 MB TIF) [file pgen.1001233.s001.tif]

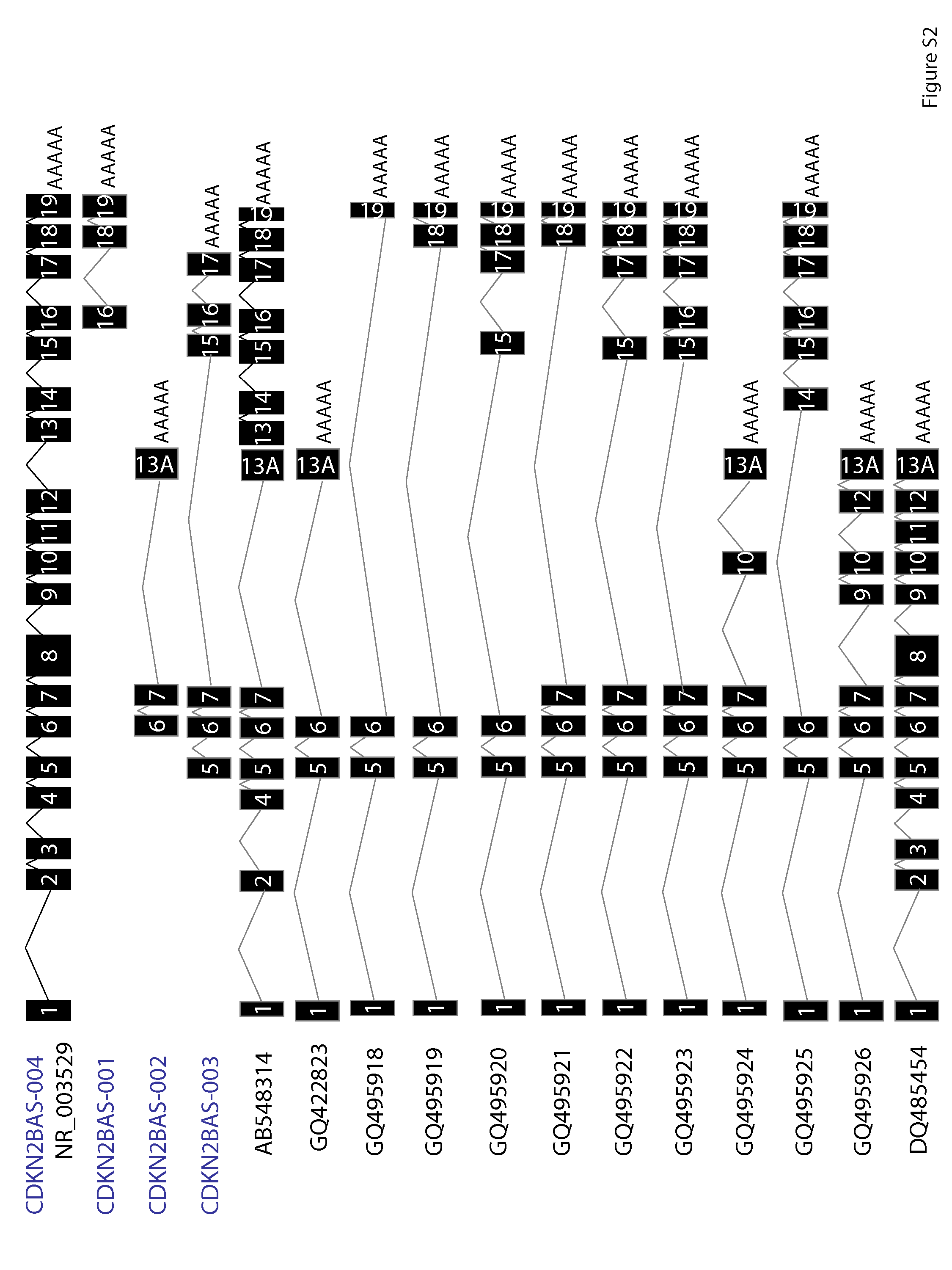

Supplement: Figure S2 — Schematic of previously reported ANRIL variants. All Ensembl (blue) and GenBank (black) records for ANRIL (CDKN2BAS) are shown. Some sequences are derived from cDNA sequencing whereas others were inferred by EST assembly. (0.64 MB TIF) [file pgen.1001233.s002.tif]

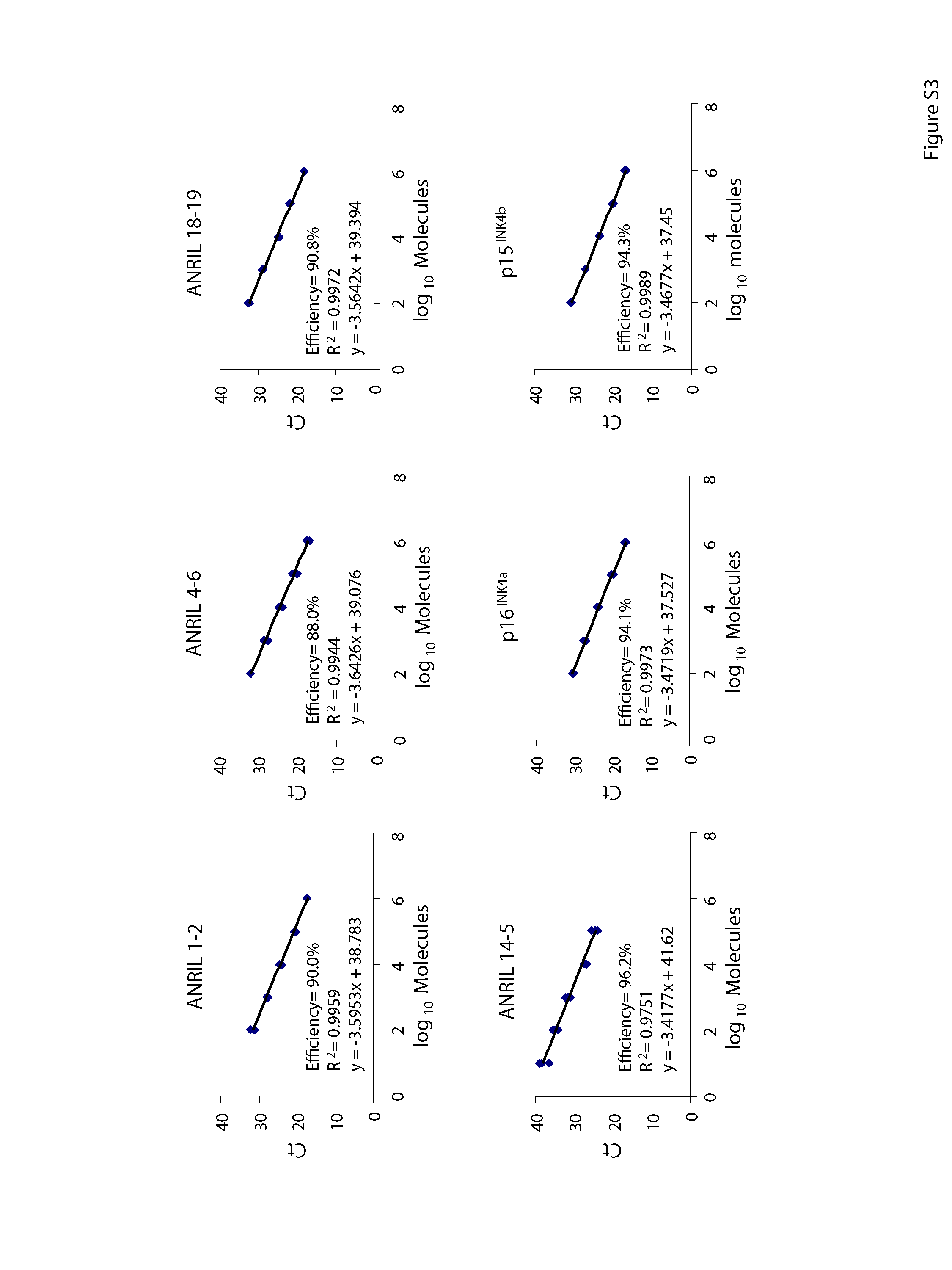

Supplement: Figure S3 — Efficiency curves for Taqman probe strategies. Cloned cDNAs or PCR products corresponding to the Taqman target sequences were linearized and quantified. For each realtime PCR run, a standard curve of 5 independent dilutions was run in triplicate. Primer efficiency was calculated using the formula: Efficiency = 10∧(-1/slope)-1. Shown are representative graphs from individual experiments. (0.52 MB TIF) [file pgen.1001233.s003.tif]

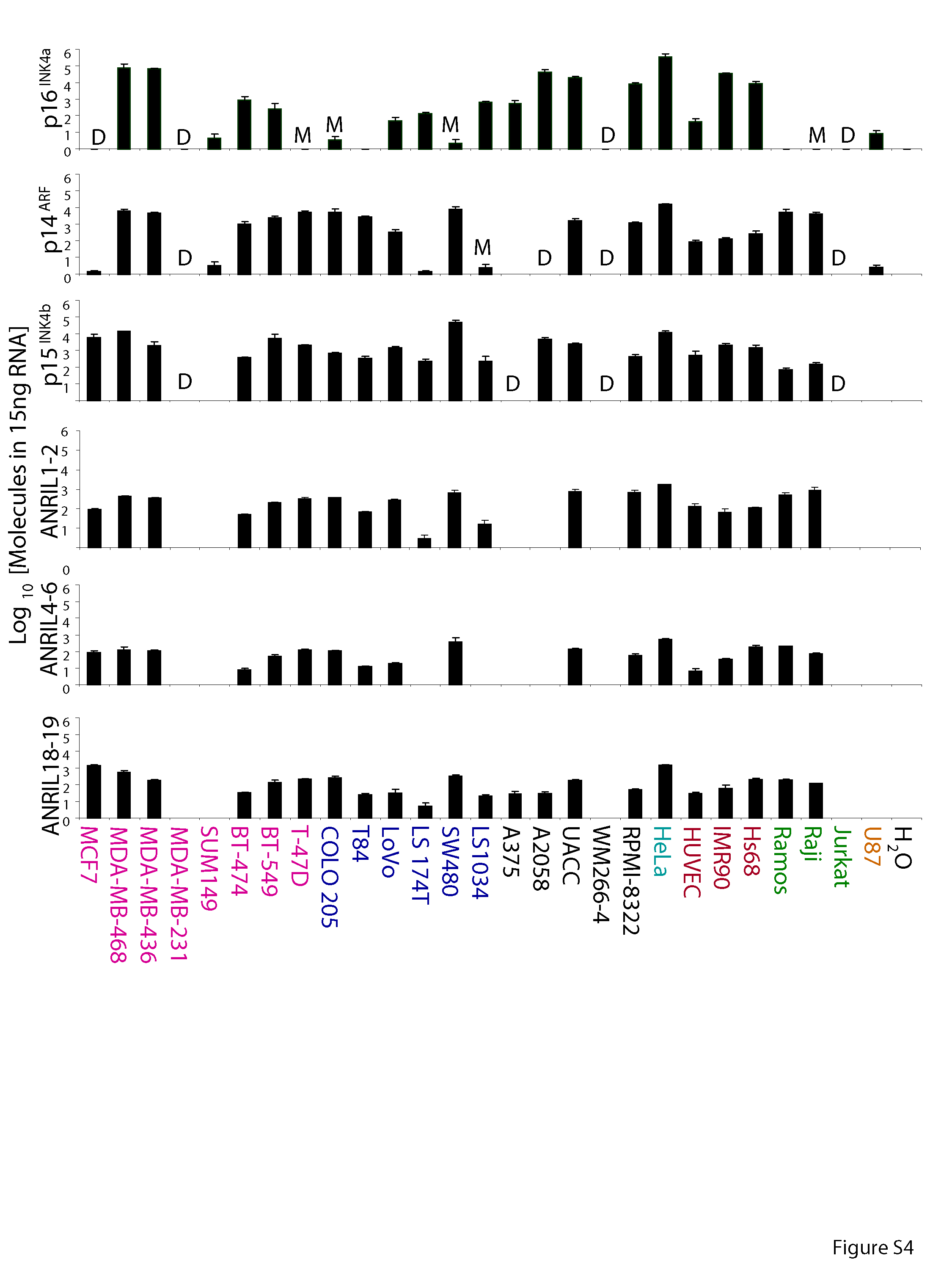

Supplement: Figure S4 — Expression of 9p21 transcripts in transformed and non-transformed cell lines. As described in Figure 1B, RNA was harvested, reverse transcribed and quantitative real-time PCR performed. Bars represent the log10 of the average number of molecules detected. The error bars denote the standard deviation between three replicates. The letter ‘D’ denotes deletion events previously reported in the literature. ‘M’ indicates gene methylation. Breast cancer are shown in pink, colorectal cancers in blue, melanomas in black, hematological malignancies in green, cervical carcinomas in turquoise, glioblastomas in gold and nontransformed cells in red. (0.58 MB TIF) [file pgen.1001233.s004.tif]

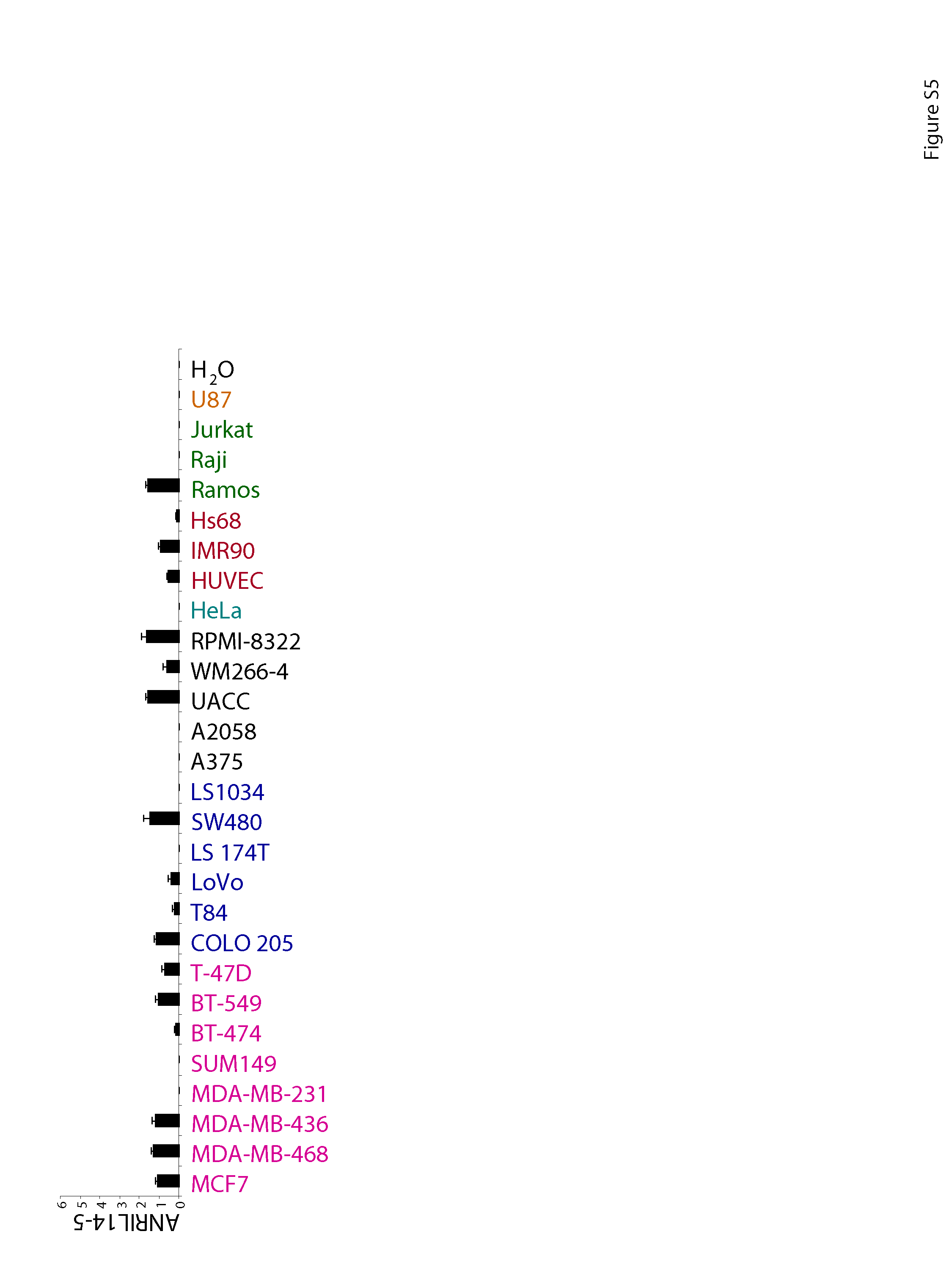

Supplement: Figure S5 — ANRIL14-5 expression is detected in a wide variety of cell types. cDNA generated as described in Figure 1B was assayed for ANRIL14-5 expression using the Taqman strategy shown in Figure 3A. Bars represent the log10 of the average number of molecules detected. The error bars denote the standard deviation between three replicates. Cell lines are color coded as in Figure S4. (0.50 MB TIF) [file pgen.1001233.s005.tif]

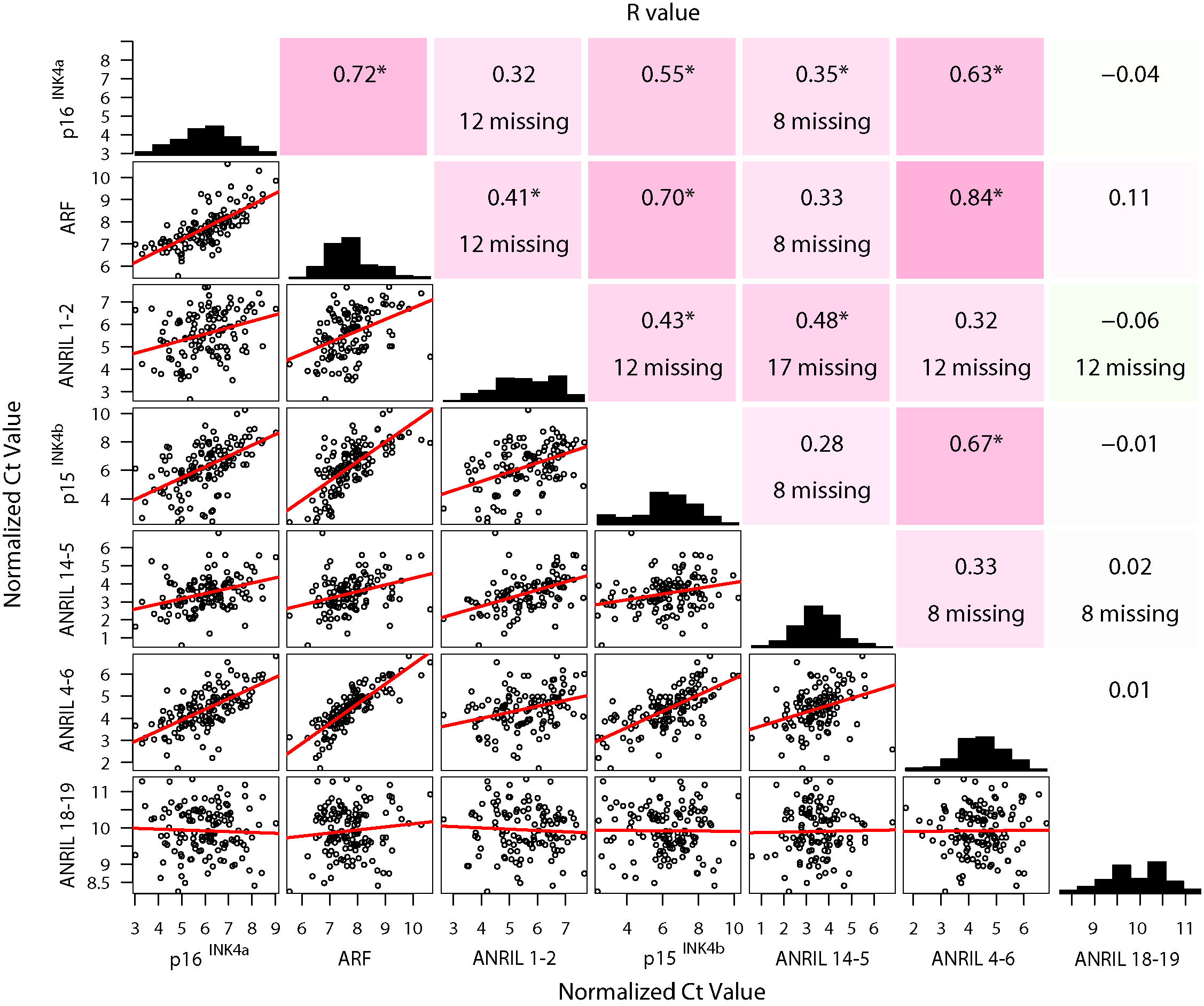

Supplement: Figure S6 — Correlation of ANRIL and INK4/ARF expression in primary peripheral blood T-lymphocytes. Diagram depicting the correlations between 9p21 transcripts in primary peripheral blood T-lymphocytes from 106 patients. Taqman anlaysis of ANRIL and INK4/ARF transcripts was conducted and normalized as described in Materials and Methods. Data for ANRIL4-6, p16INK4a, p15INK4b and ARF expression were previously reported [42]. Scatter plots, below the diagonal, show the relationships between all pairs of transcripts on a log2 scale. Linear regression is depicted in red. Boxes above the diagonal list and are color coded by r-value. A star (*) indicates significant associations (p<0.05). Histograms along the diagonal show the distribution of expression for each transcript assayed. Due to limitations in sample availability, ANRIL 1-2 and ANRIL 14-5 levels were not determined for several individuals as indicated (n = 94 and 98, respectively). (1.55 MB TIF) [file pgen.1001233.s006.tif]

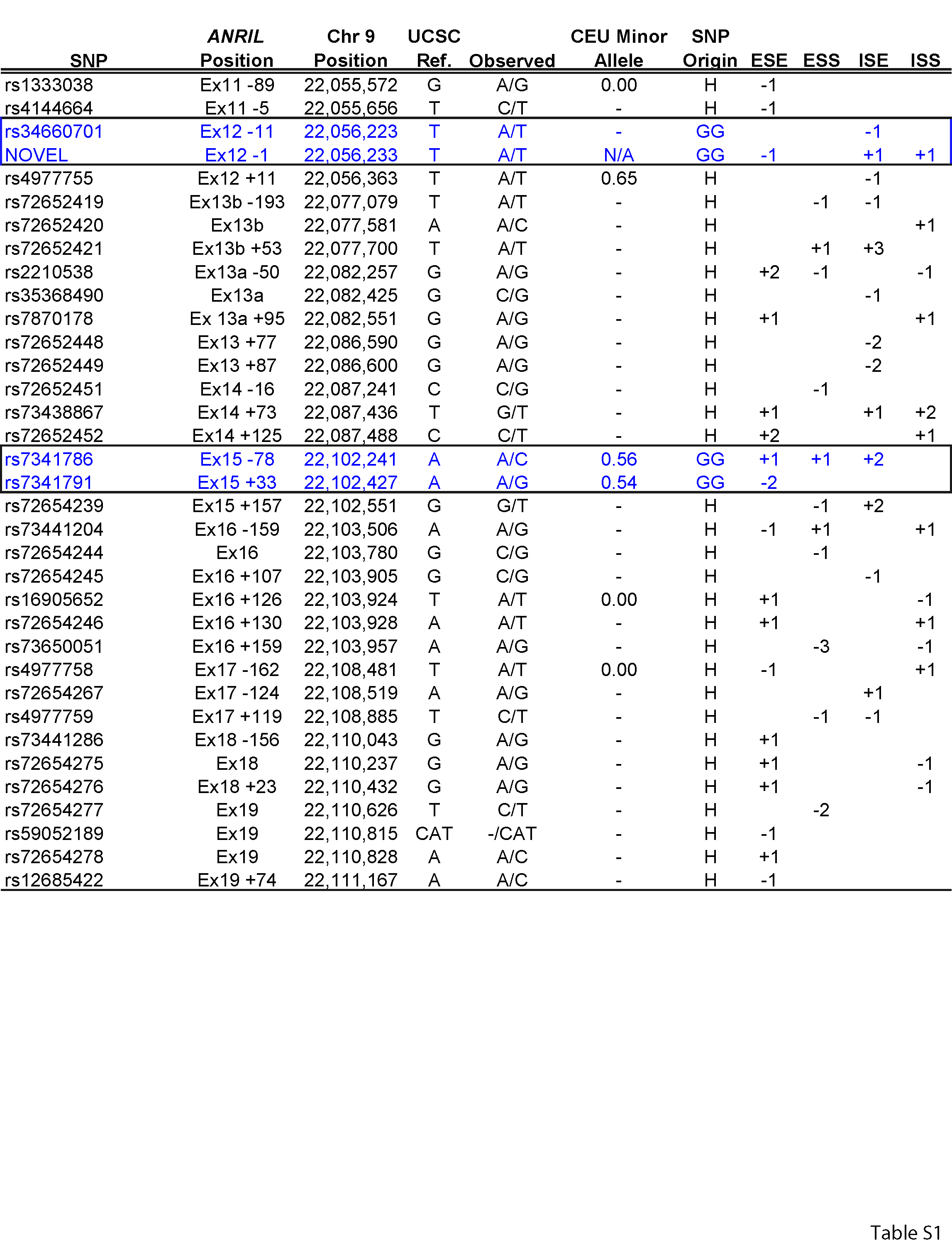

Supplement: Table S1 — Splice site analysis of polymorphisms in the ASVD risk interval near ANRIL exon-intron boundaries. SNPs within 200 bp of an ANRIL inton-exon boundary were analyzed for their effects on putative exon splicing enhancer (ESE), exon splicing silencer (ESS), intron splicing enhancer (ISE), and intron splicing silencer (ISS) sequences as described ([54], [55] and Z. Wang unpublished data). A score of -1 indicates that the minor allele destroys one cis-element and +1 indicates that the minor allele creates one cis-element. SNPs identified as unique to the AA or GG samples using sequence capture are shown in blue. Those identified in the HapMap database are depicted in black. The position of each intronic SNP relative to the nearest ANRIL exon is given under the ‘ANRIL Position’ column. Exonic SNPs in this column, list the exon in which they occur. If available, the minor allele frequency from Utah residents with ancestry from northern and western Europe (HapMap3, CEU) is given in the ‘CEU Minor Allele’ column. H- Hapmap3; GG- individuals homozygous for the ‘G’ allele at rs10757278. (0.59 MB TIF) [file pgen.1001233.s007.tif]

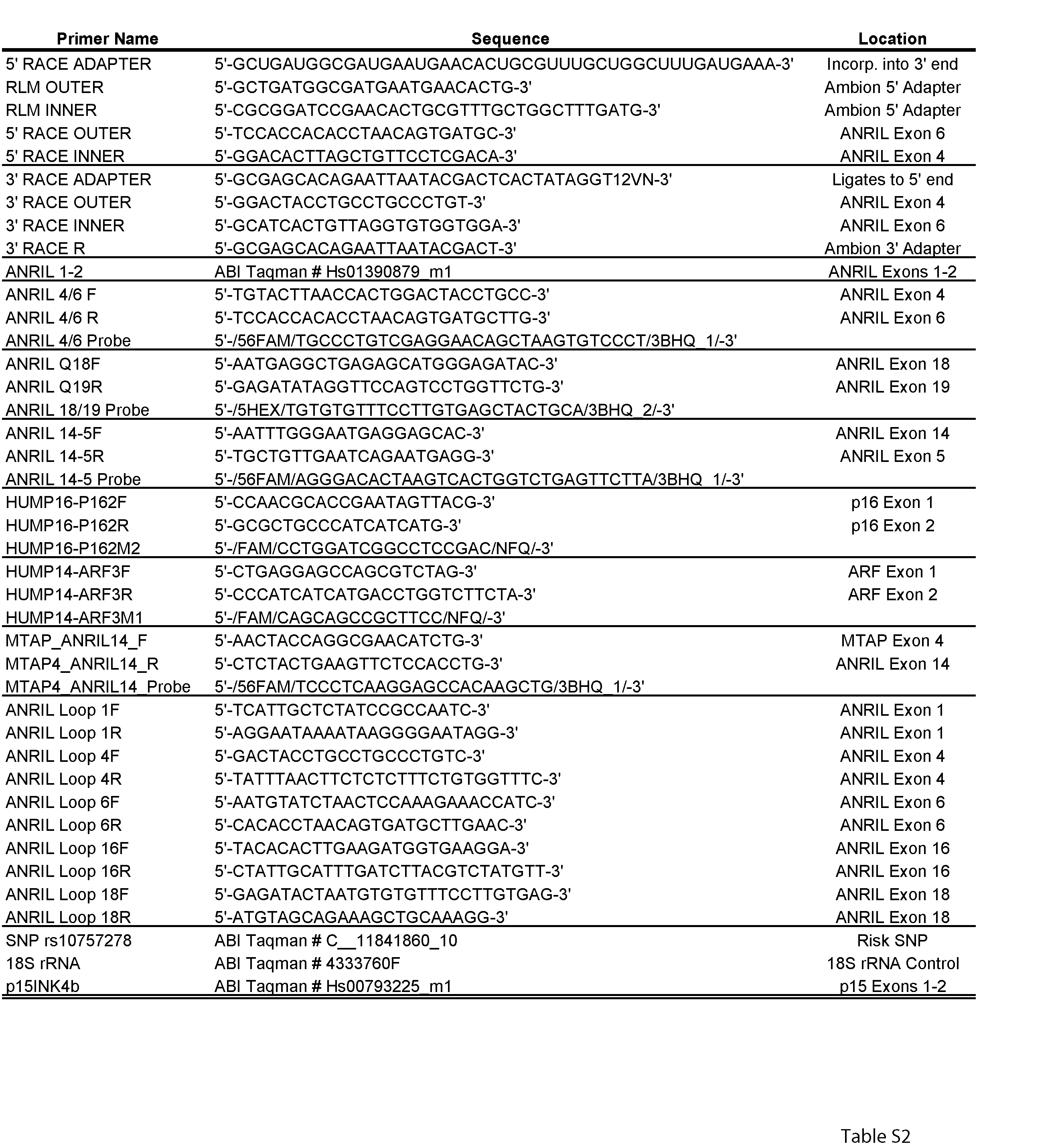

Supplement: Table S2 — Primers used for RACE, Taqman, and PCR analysis. (0.67 MB TIF) [file pgen.1001233.s008.tif]
